# Hydrologic Alterations from Climate Change Inform Assessment of Ecological Risk to Pacific Salmon in Bristol Bay, Alaska

**DOI:** 10.1371/journal.pone.0143905

**Published:** 2015-12-08

**Authors:** Cameron Wobus, Robert Prucha, David Albert, Christine Woll, Maria Loinaz, Russell Jones

**Affiliations:** 1 Abt Associates, Boulder, Colorado, United States of America; 2 Integrated Hydro Systems, Golden, Colorado, United States of America; 3 The Nature Conservancy, Juneau, Alaska, United States of America; 4 A.D.A. Engineering, Inc., Tampa, Florida, United States of America; Universidade de Aveiro, PORTUGAL

## Abstract

We developed an integrated hydrologic model of the upper Nushagak and Kvichak watersheds in the Bristol Bay region of southwestern Alaska, a region under substantial development pressure from large-scale copper mining. We incorporated climate change scenarios into this model to evaluate how hydrologic regimes and stream temperatures might change in a future climate, and to summarize indicators of hydrologic alteration that are relevant to salmon habitat ecology and life history. Model simulations project substantial changes in mean winter flow, peak flow dates, and water temperature by 2100. In particular, we find that annual hydrographs will no longer be dominated by a single spring thaw event, but will instead be characterized by numerous high flow events throughout the winter. Stream temperatures increase in all future scenarios, although these temperature increases are moderated relative to air temperatures by cool baseflow inputs during the summer months. Projected changes to flow and stream temperature could influence salmon through alterations in the suitability of spawning gravels, changes in the duration of incubation, increased growth during juvenile stages, and increased exposure to chronic and acute temperature stress. These climate-modulated changes represent a shifting baseline in salmon habitat quality and quantity in the future, and an important consideration to adequately assess the types and magnitude of risks associated with proposed large-scale mining in the region.

## Introduction

The Bristol Bay region of southwestern Alaska supports one of the world’s largest wild salmon fisheries, supplying over 50% of wild sockeye salmon catches worldwide [1]. The region also has substantial mineral resources [2], and is facing increasing pressure for development of these resources. Ongoing debate about the future of this region has focused on the potential impacts of mining on the fishery, recently culminating in a draft ruling by the U.S. Environmental Protection Agency that would limit mining in the Bristol Bay region [3]. While hydrologic alterations due to mining could be substantial [3,4], salmon habitat quality could also be significantly modified by climate change over timescales that are relevant to planning for any large-scale mining activities. In this study, we present results from a spatially explicit model of climate-driven changes to freshwater ecosystems, as a backdrop for assessment of ecological risks to salmon associated with large-scale mining.

In the freshwater environment, hydrologic variability and the salmon life cycle are closely linked, so that climate-induced changes in hydrologic regimes are likely to influence salmon productivity. In cold environments, climate change is projected to alter seasonal cycles of snow accumulation and melt [5,6], affecting seasonal habitat quantity and quality and potentially migratory timing [7]. Stream temperatures are also likely to increase in the future, which could shorten salmon egg incubation times and increase juvenile growth rates [8], as well as reduce thermal habitat suitability and survival [9,10]. Since climate changes at high latitudes are expected to be amplified relative to other parts of the world, all of these potential changes could be both more dramatic and more rapid in Alaska than at lower latitudes [11]. However, the natural diversity of salmon habitat and populations in this region represent a critical asset that could allow salmon to adapt to changing conditions over time [12].

**Fig 1:**
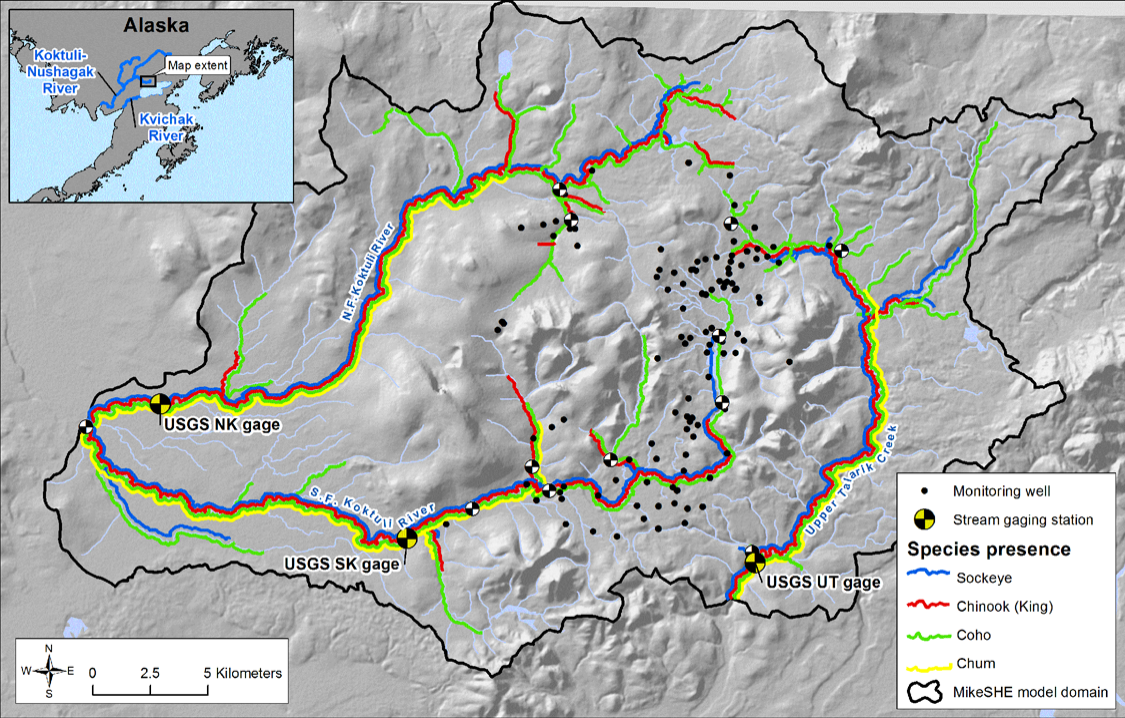
Site Location Map. Salmon presence as documented in the anadromous waters catalog [14].

We use an integrated hydrologic model and a range of future climate scenarios to characterize changes in the hydrology of the upper Nushagak (North and South Fork Koktuli) and Kvichak (Upper Talarik) rivers, two major salmon systems that drain into Bristol Bay (Fig 1). Using results from our hydrologic model, we broadly follow the Indicators of Hydrologic Alteration (IHA) approach of Richter et al. [13] to focus on changes in hydrologic indicators that are relevant to salmon habitat quality. These indicators include the magnitude, timing, and variability of flow, as well as average and extreme stream temperatures. The integration of hydrologic modeling results with the IHA approach provides a framework for understanding climate change impacts on habitat quality that is both quantitative and spatially explicit, and can help to inform risks from other stressors including mining. While this model was developed for the Bristol Bay region, the modeling framework we describe could be applied in other settings where climate-modulated changes in habitat quality need to be quantified.

## Background and Methods

### Site Description

The study region is located in southwestern Alaska, and has a sub-arctic maritime climate. Because of the mineral resources in the area, the baseline hydrology and ecology of the system have been well-characterized through extensive study by both mine proponents and by the U.S. Environmental Protection Agency [3,15,16]. The physiography of the study area is characterized by low relief terrain with exposed bedrock on ridges and hilltops, and thick, coarse-grained glacial outwash filling valleys to depths of up to 100 m [3,16]. The relatively low relief and complex glacial deposits have created a hydrologic system in which groundwater and surface water are closely linked: precipitation and stream water infiltrate into the coarse gravel in the upper reaches of the watershed, returning to the rivers at points downstream [3,15]. This groundwater input moderates stream temperatures throughout the year, provides a steady baseflow to the rivers through the relatively dry winter and summer months, and maintains open water reaches in winter when the majority of the landscape is frozen [3,15].

The annual hydrograph at the site is dominated by high flows in the fall and spring, with relatively low flows during the summer and winter [3,15]. High flows in the spring are related to ice breakup and snowmelt, and create a spring freshet that consistently occurs between mid-May and early June. High flows in the fall are driven by frequent fall storms, which can fall either as rain or snow [15]. Winter stream flows are consistently low, fed primarily by groundwater when the majority of precipitation is frozen and is stored as snow on the landscape. Summer flows are also relatively low, punctuated by frequent summer rainstorms.

Salmon are present throughout the hydrologic network, and they utilize both mainstem and tributary habitats for spawning and rearing (Fig 1). The system supports sockeye (*Oncorhynchus nerka)*, Chinook (*O*. *tshawytscha)*, coho (*O*. *kisutch)* and chum salmon (*O*. *keta)*.

### Model Description

We built a hydrologic model for this study using MIKE SHE/MIKE 11 [17], by adapting a previous model developed to characterize potential hydrologic and water quality alterations due to large-scale mining scenarios [4]. MIKE SHE is a fully distributed parameter, integrated hydrologic code that simulates the flow of water within and among surface water, groundwater, and the unsaturated zone. Continuous flows within the hydrologic system are driven by external atmospheric conditions, including precipitation, air temperature, and evapotranspiration. Using a modified degree-day snowmelt method, the code simulates snow accumulation if air temperatures fall below a freezing threshold (typically 0°F), and it also simulates snowmelt processes including evaporation (sublimation and wet-snow evaporation), rain-on-snow, changes in wet and dry snow storage, and refreezing of wet snow. For this effort, we also implemented a heat balance algorithm built using the DHI ECO Lab module to simulate stream temperatures throughout the model domain at 15-minute timesteps [7,18]. Thus our model estimates spatially and temporally distributed stream flow and stream temperature throughout the model domain. We defined the model domain to be large enough to prevent influence of external boundary conditions (e.g., stream and groundwater flows) on internal calculations, but small enough to maximize the use of publicly available baseline data collected by the U.S. Geological Survey (USGS) and the Pebble Limited Partnership (PLP) over the past decade [15,16]. Physical data used in the model included vegetation cover [19] and associated shading parameters [20], soil types [21], and the distribution and thickness of coarse glacial outwash material [16]. Calibration data included stream discharge records from USGS and PLP gages, groundwater elevations measured by PLP at approximately 200 wells between 2004 and 2007 [15], and stream and groundwater temperatures measured both synoptically and as timeseries across a set of monitoring wells and gages throughout the model domain. Detailed description of the hydrologic model setup and parameterizations, and flow calibration results can be found in Wobus et al. [4]. The heat balance algorithm and parameters used to estimate stream temperatures in the model are described in Loinaz et al. [18]. The locations of hydrologic monitoring sites used in the model calibration are shown in Fig 1.

We developed this model to be as simple as possible while honoring all available physical data [22]. Using the constraints available from measured and literature-based hydrologic and land cover data, we calibrated our final set of model parameters primarily against observed stream temperatures, the magnitude and timing of spring runoff and baseflows, and the observed distribution of groundwater elevations throughout the model domain [4]. We stress that the goal of this study was not to perfectly reproduce baseline conditions in this system; fine scale spatial variability in surface topography, vegetation cover, subsurface geology, and other key variables cannot be known well enough to support such a goal over ~800 km^2^ of remote southwestern Alaska. Furthermore, the gridded climate data for the region cannot perfectly capture the spatial and temporal sequence of storm events that drive the hydrologic system. As described below, however, our calibrated model captures the fundamental hydrologic and heat balance characteristics of the natural, undisturbed system, and serves as a launching point to examine the magnitude of change under future climate scenarios.

### Baseline and Future Climate Simulations

**Fig 2:**
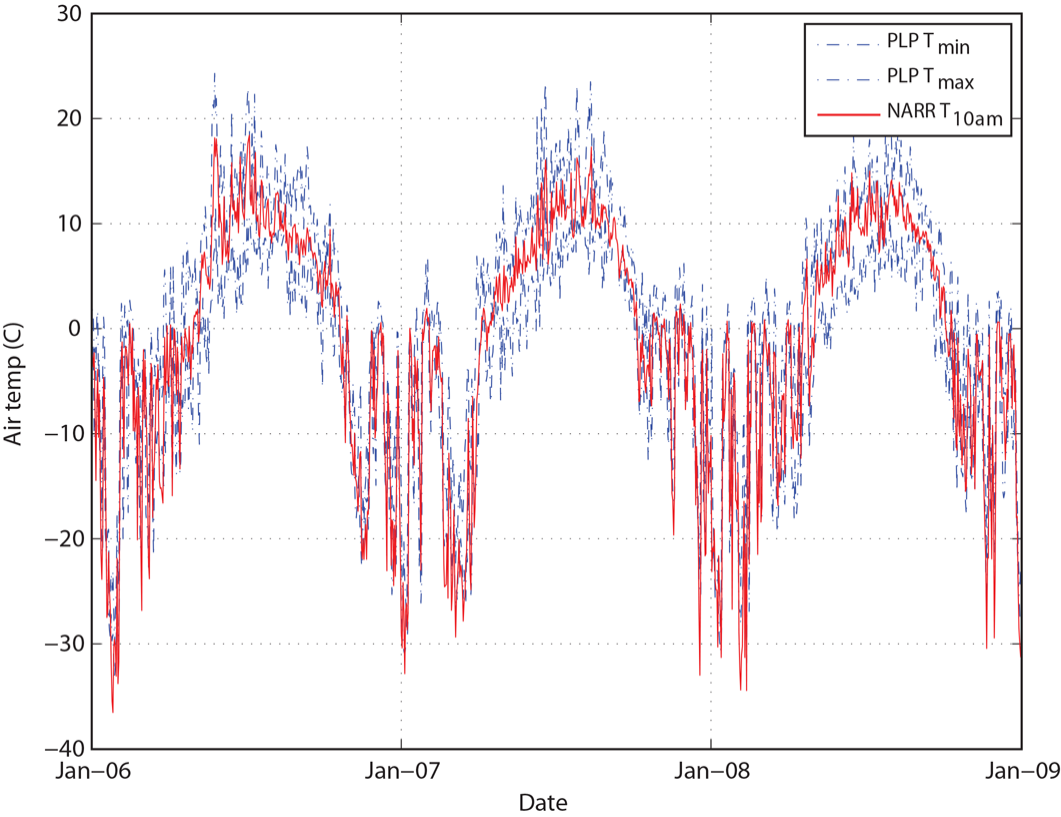
Comparison of NARR vs. Measured Temperature Data over the Period 2006–2009.

Available site meteorological records are inconsistent in space and time, and lack many of the physical variables necessary to drive a fully coupled hydrologic and heat balance model. As a result, we utilized the 3-hourly North American Regional Reanalysis (NARR) product as the meteorological forcing for our hydrologic model. The NARR data include all of the key variables driving the hydrologic cycle, including precipitation, temperature, net radiation (sum of incoming minus outgoing short- and long-wave radiation), relative humidity, and wind speed, with a 3-hour temporal resolution and a 32-km spatial resolution [23]. We extracted a 30-year timeseries of NARR data from the 32 km × 32 km cell overlying the Nushagak-Kvichak headwaters for the period of 1980 to 2009. During time periods where the NARR data overlap with site-specific information, comparisons of the two datasets indicate that the NARR data generally captures the magnitude and variability of precipitation and temperatures observed in the natural system (e.g., Fig 2) [4].

**Fig 3:**
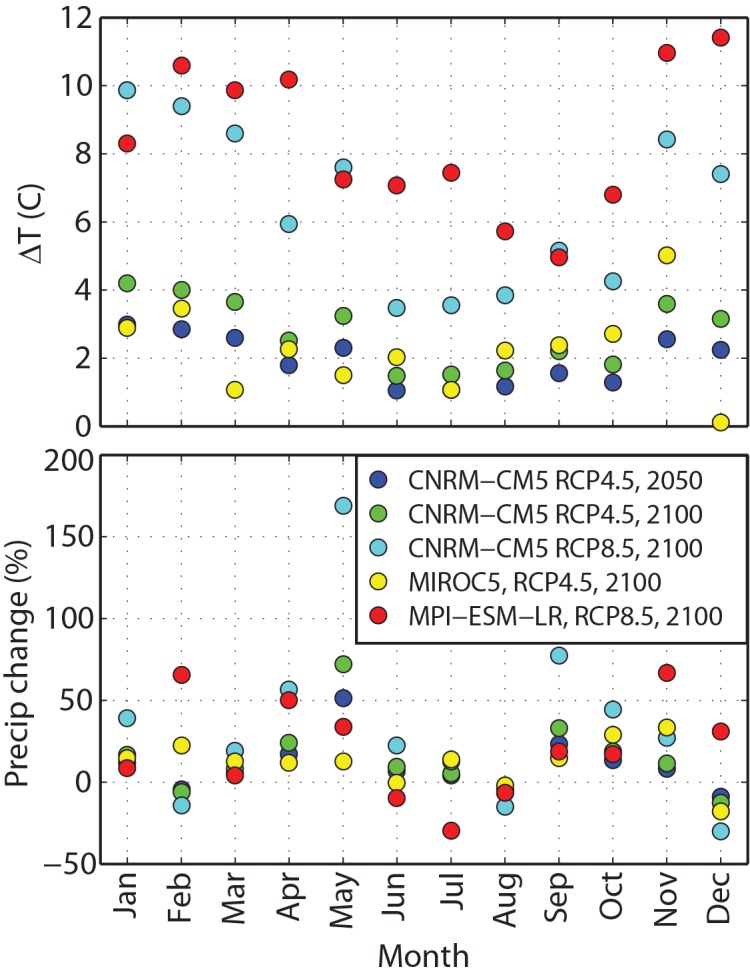
Monthly Changes in Temperature and Precipitation Projected for the 5 Models Used in Climate Change Simulations.

Walsh et al. [24] compared the performance of 15 climate models based on root-mean-square errors relative to observed climatology in Alaska over the second half of the 20th century. Using this group of GCMs as a starting point, we developed future climate scenarios from the suite of five models identified as best-suited to simulating baseline climate conditions in Alaska. Using these models, we bracketed future temperature changes by selecting two greenhouse gas emissions pathways. The lower pathway (RCP 4.5) represents stabilization of atmospheric CO_2_ by the end of the century and a net increase in radiative forcing of 4.5 W/m^2^ relative to pre-industrial times. The higher pathway (RCP 8.5) assumes continued growth of CO_2_ emissions beyond 2100 and an increase in radiative forcing of 8.5 W/m^2^ compared to pre-industrial times [25]. Of the five models (CNRM-CM5, GFDL-CM3, HADCM3, MIROC5 and MPI-ESM-LR) the HADCM3 model was a clear outlier in its projections of future climate changes, and was rejected (Table 1). From the remaining four models, we then bracketed potential future conditions by choosing the two model-emissions pairs projecting maximum (MPI-ESM-LR, RCP8.5) and minimum (MIROC5, RCP4.5) changes in annual average temperature at the site in 2100. We also chose an intermediate model (CNRM-CM5), which we used to simulate both a high and a low emissions pathway, as well as two time periods for the lower emissions pathway (2050 and 2100). We then extracted monthly changes in temperature and precipitation from the final five model-scenario pairs (Fig 3).

**Table 1:** Average Annual Projected Changes in Temperature and Precipitation for 5 Models Initially Considered for Simulations. RCP 4.5 and RCP 8.5 are two “representative concentration pathways” signifying increases in radiative forcing of +4.5 and +8.5 W/m^2^ relative to pre-industrial greenhouse gas concentrations. See text for details.

	Temperature projections				Precipitation projections			
	RCP 4.5		RCP 8.5		RCP 4.5		RCP 8.5	
Model	2050	2100	2050	2100	2050	2100	2050	2100
CNRM-CM5	1.96^a^	2.75^a^	2.68	6.46^a^	10.3%^a^	14.5%^a^	14.2%	34.0%^a^
GFDL-CM3	2.11	2.96	2.90	6.97	–	–	–	–
HADCM3	0.37	0.52	0.51	1.22	–	–	–	–
MIROC5	1.59	2.23^a^	2.18	5.24	8.6%	12.1%^a^	11.8%	28.4%
MPI-ESM-LR	2.54	3.56	3.48	8.38^a^	6.3%	8.9%	8.7%	20.9%^a^

^a^These values represent the final model runs used in simulations.

We simulated 20-year long future climate scenarios for 2050 and 2100 using the delta method, by superimposing projected monthly changes in precipitation and temperature onto the observed NARR time series [26]. The synthetic future climate dataset therefore preserves the same sequence of weather and storms as the baseline run, allowing for a direct comparison between baseline and future hydrologic and stream temperature conditions.

Groundwater is a key component of the stream flow heat balance equations in MIKE SHE, but groundwater temperatures are not explicitly calculated in the ECO Lab module. For baseline conditions, we used average monthly groundwater temperatures measured in monitoring wells to estimate the temperature of groundwater inputs to streams. For future conditions, we modified groundwater temperatures for a subset of the model runs, as described below.

Snowpack insulates aquifers from colder air temperatures in the winter, so the annual average groundwater temperature at the site of ~3°C is warmer than the average annual air temperature of -0.5°C [27]. As described below, future climate scenarios predict that winter precipitation will commonly fall as rain instead of snow, eliminating the insulating effect of snowpack on groundwater temperature. We assumed that average annual groundwater temperature tracks average annual air temperature without snowpack. However, because the current average annual temperature for groundwater is 3°C warmer than the average annual air temperature, we did not force any changes to groundwater temperature if the projected average annual air temperature was equal to or less than 3°C. Thus the three future climate scenarios based on RCP 4.5 included no adjustment to groundwater temperature. For climate scenarios that had a predicted annual average air temperature higher than the current groundwater temperature of 3°C, we assumed that groundwater temperatures equilibrated to this higher air temperature. Thus the future climate simulations for CNRM-CM5 and MPI-ESM-LR under RCP 8.5 included increases in average annual groundwater temperature of 3.2 and 4.9°C, respectively to match air temperatures. As will be shown below, many of the changes we see in flow and average stream temperatures occur across all models, and these key model results are therefore insensitive to this input assumption.

### Hydrologic Alteration Framework

The IHA framework described by Richter et al. [13] summarizes 32 hydrologic indicators that are important for the ecological function of aquatic ecosystems. These hydrologic indicators are placed into five broad groups describing the magnitude of average flow conditions, the magnitude and timing of extreme flow conditions, and the frequency of changes in flow. We used this framework as a broad guideline for our study, modified to focus on parameters that are particularly relevant to the salmon life cycle, including water temperature (Table 2).

**Table 2:** Key Hydrologic Parameters used for Quantifying Salmon Habitat Quality under Baseline and Future Climates. TDD = temperature degree days.

Parameter group	Hydrologic parameter	Ecosystem influence
**Magnitude of peak flow**	Annual maximum peak flow	Channel forming flow
		Scour of redds
**Timing of peak flow**	Julian date of peak flow	Scour of redds
		Smolt migration
**Magnitude of seasonal flow**	Mean daily summer flow (Jul. 1–Aug. 31)	Spawning and rearing habitat availability
	Mean daily winter flow (Dec. 1–Apr. 30)	Overwinter habitat quality
**Average temperatures**	TDD accumulated from peak spawning date through egg incubation	Incubation time
	Mean temperature	Juvenile growth rates
**Extreme temperatures**	Days above 20°C	Chronic temperature stress
	Days above 25°C	Acute temperature stress

We included parameters describing the magnitude and timing of peak flow due to their influence on redd (salmon spawning nest) scour, substrate size, and smolt migration. High flows during winter can scour eggs within the gravel, decreasing egg survival [6,28]. High spring peak flows allow for easier outmigration for salmon smolts, which can improve survival [29–32]. Adequate summer flows are also needed to maintain available spawning and rearing habitats [29,33], and winter flows are required to provide an abundance of slow-moving backwater habitats for overwintering Chinook, river-type sockeye, and coho salmon [29].

We also included parameters related to average annual and seasonal water temperature, because water temperature is directly linked to egg incubation time and juvenile salmon growth rates [34,35]. For example, based on laboratory observations salmon eggs require approximately 600 TDD (°C·day) to mature and hatch [8]. Peak water temperatures are also relevant to salmon habitat quality; prolonged stream temperatures above 20°C can cause chronic temperature-related stress to salmon, and temperatures above 25°C can cause acute temperature stress and mortality [10,36,37].

We summarized changes in each of these parameters by comparing future daily flows and water temperature to baseline conditions across all 546 computational nodes where flows and water temperatures are simulated within the model domain.

## Results

### Model Calibration

**Fig 4:**
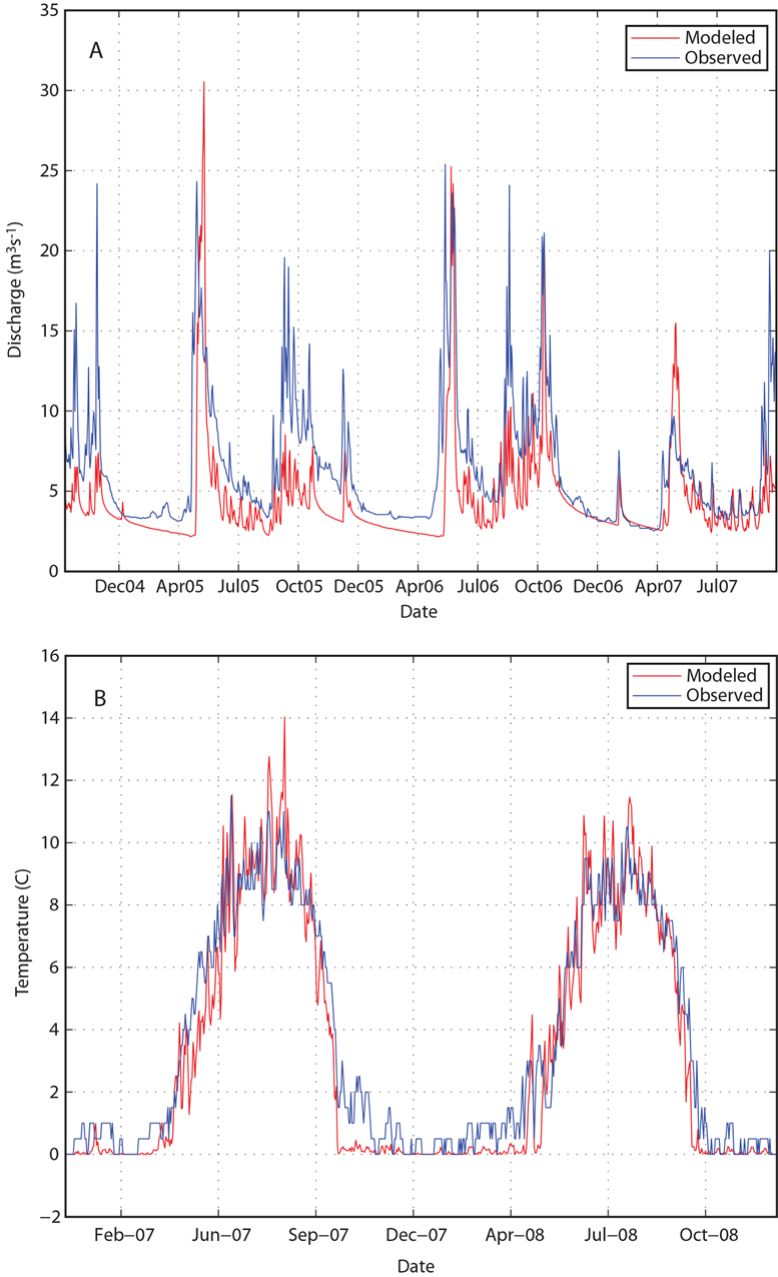
Modeled vs. Observed (a) Hydrograph and (b) Stream Temperatures at USGS Gage Site on Upper Talarik Creek (See Map for Location).

There are fifteen stream gaging stations within the model domain, and all of these sites have at least two years of continuous data (Table 3). Correlation coefficients for nearly all of the gage sites were greater than 0.6 for our calibrated model. The timing of simulated peak spring runoff was typically within a week of observed runoff; for example, at a representative USGS gaging site on Upper Talarik Creek, the model simulates the timing of peak spring runoff to within 3–5 days of the observed spring peak (Fig 4A). Flow calibration plots for all fifteen gages showed similar behavior (Figs A-N in S1 File), indicating that the model parameterization of temperature-modulated snow accumulation and melt is robust.

**Table 3:** Flow and Temperature Calibration Statistics for All Gage Sites Modeled. RMSE = root mean square mean error (flow values in m^3^/s; temperature values in°C); R = correlation coefficient. Note that continuous temperature measurements were available only at the three USGS gage sites.

Gage Site	Flow Calibration		Temperature Calibration	
	RMSE	R	RMSE	R
UTK15300250	3.81	0.65	1.20	0.95
SFK15302200	5.24	0.61	1.62	0.95
NFK15302250	6.14	0.71	1.72	0.94
SK100A	6.57	0.72	–	–
SK100B1	3.35	0.74	–	–
SK100C	1.39	0.75	–	–
SK100F	0.73	0.71	–	–
SK100G	0.35	0.63	–	–
SK119A	1.30	0.66	–	–
SK124A	0.78	0.59	–	–
UT100D	0.62	0.65	–	–
UT100E	0.18	0.71	–	–
UT119A	0.18	0.31	–	–
NK100C	1.07	0.67	–	–
NK119A	0.69	0.66	–	–

The magnitudes of the peak flows were not as well matched between the modeled and observed data as the timing of peak flows (Figs A-N in S1 File). At the Upper Talarik site (Fig 4A), a representative site, the difference between simulated and observed peak spring flow is often 20–50%, and peak flows in the fall are systematically under-simulated by the model. Efforts to improve the fit to these seasonal flow magnitudes by adjusting parameters such as evapotranspiration or infiltration rates resulted in hydrographs that rose and fell too rapidly, and baseflows that were too low. We hypothesize that differences in the magnitudes of observed and simulated flow relate to differences between actual precipitation and the precipitation simulated by the NARR product [23,38]. However, given the general agreement between timing of observed and simulated hydrographs, and our objective of simulating flow alterations due to future climate change, the hydrographs simulated by the model provide a reasonable representation of baseline hydrologic conditions.

Monthly synoptic stream temperature measurements are available at approximately 73 locations between 2004 and 2010, but continuous temperature measurements are available only at the three USGS gaging stations. Table 3 includes the temperature calibration metrics for these three gage sites, and Fig 4B shows a timeseries of modeled and observed temperatures at the USGS Upper Talarik gage site. At this and the other USGS gage sites (Figs O and P in S1 File) simulated and observed stream temperatures range from 0°C in winter to approximately 10–12°C in summer, and the model captures the seasonality of temperature changes between these extremes. Accumulated TDD during the fall-winter incubation period also agree closely, as described in more detail below.

### Climate Change Simulations

#### Changes in flow

**Fig 5:**
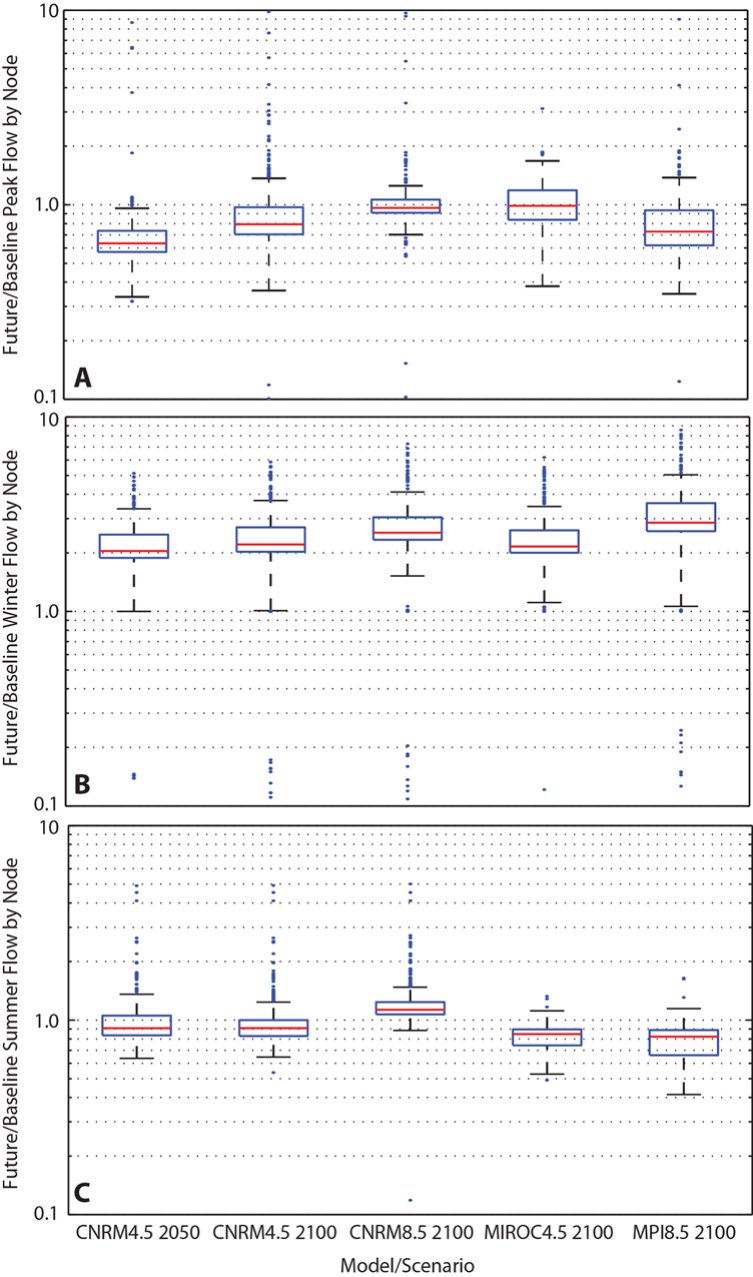
Summary of Flow Changes in Future Climate Scenarios. A) Ratio of peak flow, by model node, in future scenarios to peak flow in baseline scenarios. Peak flow values are calculated without respect to date of peak flow. B) Ratio of average winter flow, by model node, in future scenarios to average winter flow in baseline. C) Ratio of average summer flow, by model node, in future scenarios to average summer flow in baseline.

**Fig 6:**
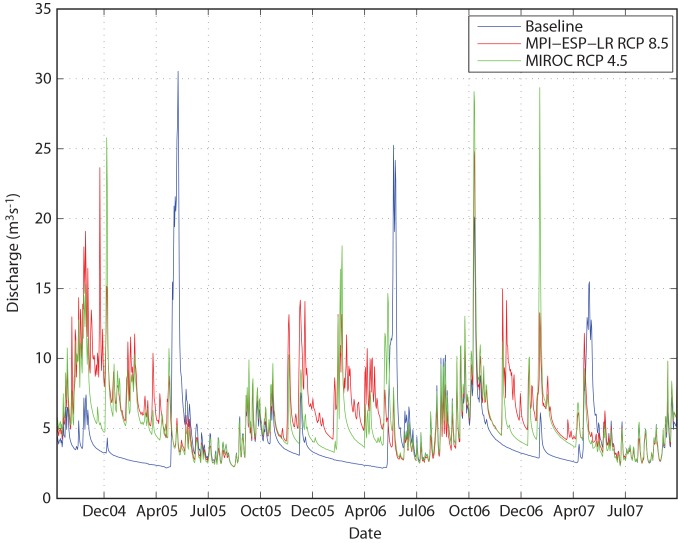
Changes in Hydrograph for Upper Talarik Creek Gage Site in 2100, for Lowest (MIROC, Green Line) and Highest (MPI, Red Line) Temperature Scenarios. Note the loss of the spring freshet in both future climate simulations.

Peak annual flow decreases between 20–40% in the two CNRM RCP 4.5 simulations and in the MPI RCP 8.5 scenario, but is within 10% of baseline in the other two simulations (Fig 5A). Thus climate change does not appear to lead to significant changes in peak annual flow magnitudes. However, these broad similarities in peak annual flow magnitudes mask significant changes in the timing of annual flow peaks: as shown in Fig 6, the peak flow under the baseline scenario consistently occurs between late May and early June. In the future simulations, the peak flow can occur in virtually any month of the year, depending on the timing of individual rain storms.

A related change in the hydrograph is an approximate doubling of average winter (DJF) flow (Fig 5B). Because winter precipitation is more likely to fall as rain in the future, winter flows are characterized by episodic runoff events, rather than the low, steady baseflow that currently characterizes this system (Fig 6). Changes in summer flows (JJA) are neither as consistent nor as large as changes in winter flows. Simulated summer flows are typically within 10–15% of baseline conditions across all future simulations (Fig 5C, Fig 6).

The flow alterations described above occur regardless of which future climate scenario is chosen: both the “hot” (MPI-ESP-LR RCP 8.5) and “cool” (MIROC5 RCP 4.5) endmember scenarios show similar behavior in 2100 (Fig 6). In both cases, the spring freshet that currently occurs consistently with melting of winter snowpack in late May is partially to completely lost, and all of the moisture that is stored in snowpack under the baseline model is instead released in a high frequency series of smaller runoff events throughout the winter.

**Fig 7:**
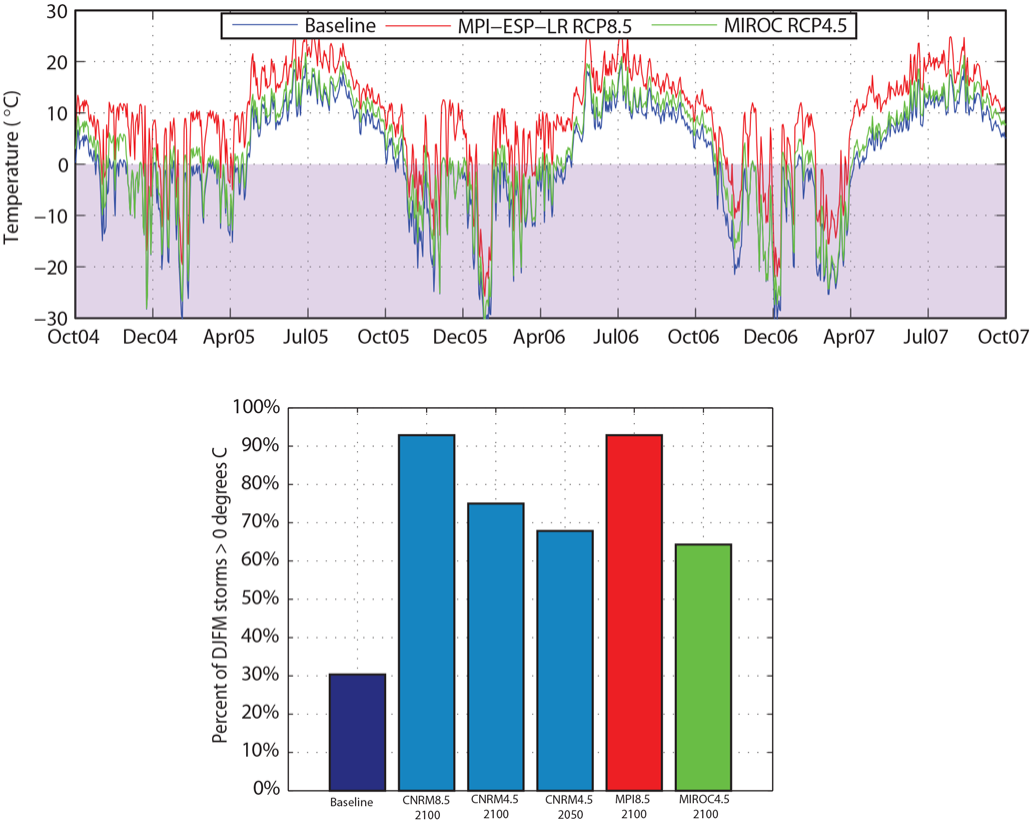
Summary of Baseline and Projected Daily Temperatures (a), and Fraction of Winter Storms Occurring when Temperatures are Above Freezing (b). Purple shading in (a) highlights temperatures below freezing.

These changes in frequency and magnitude of seasonal flow events can be related to increases in average annual and monthly temperatures. Under current climate conditions, precipitation from winter storms most commonly falls as snow, and consistently cold temperatures through the winter allow this snowpack to build up through the season (Fig 7A). This accumulated snow is released as spring temperatures rise, resulting in a spring freshet that consistently occurs in late May–early June. Among future climate scenarios, even in the “coolest” scenario for 2100 (MIROC5, RCP 4.5), monthly average winter temperatures increase by 2–4°C (Fig 3). Assuming the same distribution of storms but higher average monthly temperatures, under these conditions more than half of winter storms are projected to occur when the air temperature is above freezing (Fig 7B).

#### Changes in stream temperature

**Fig 8:**
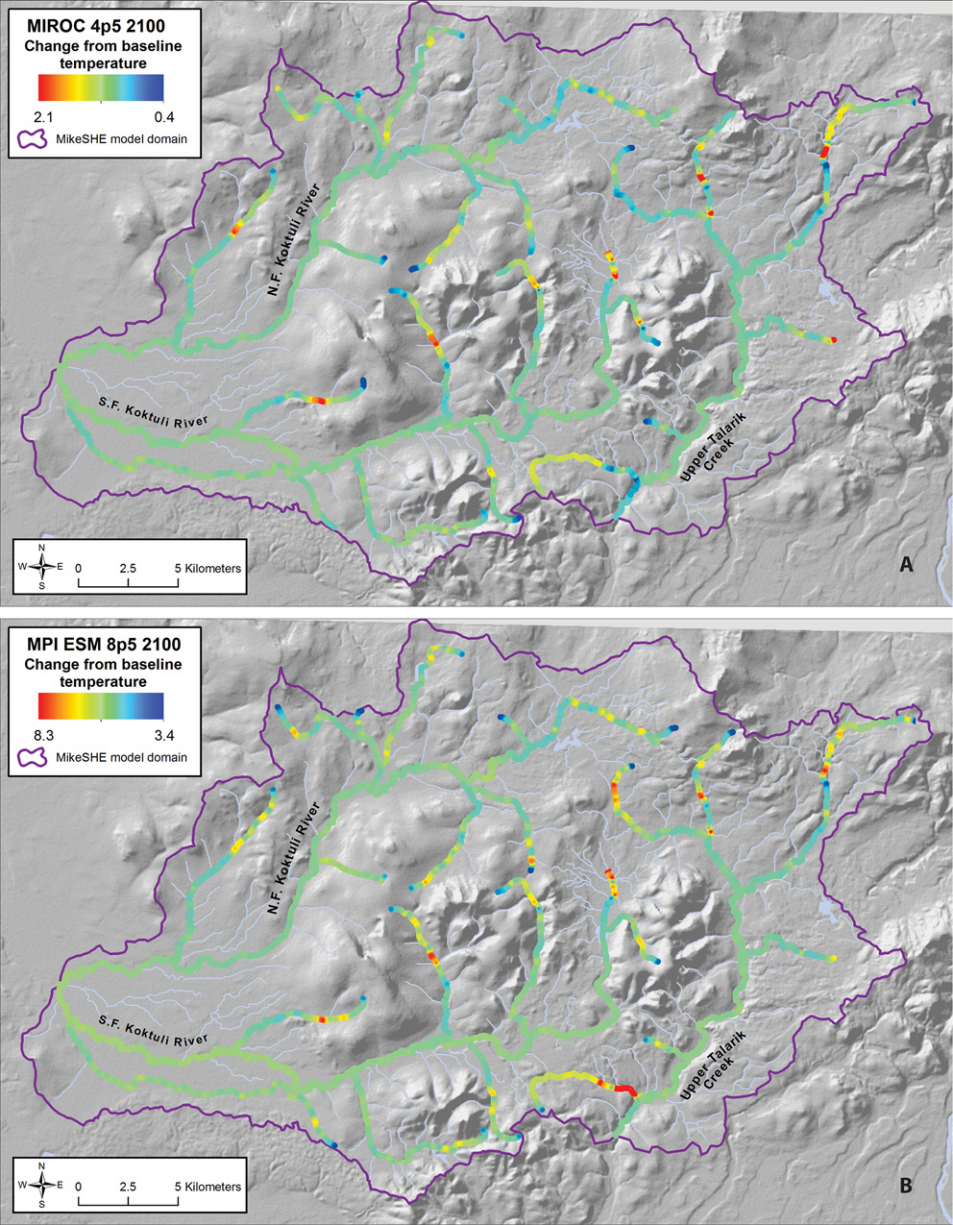
Modeled Change in Annual Average Stream Temperatures in 2100 for (a) “Cool” Scenario (CNRM-CM5, RCP 4.5) and (b) “Hot” Scenario (MPI-ESM-8.5 RCP 8.5).

**Fig 9:**
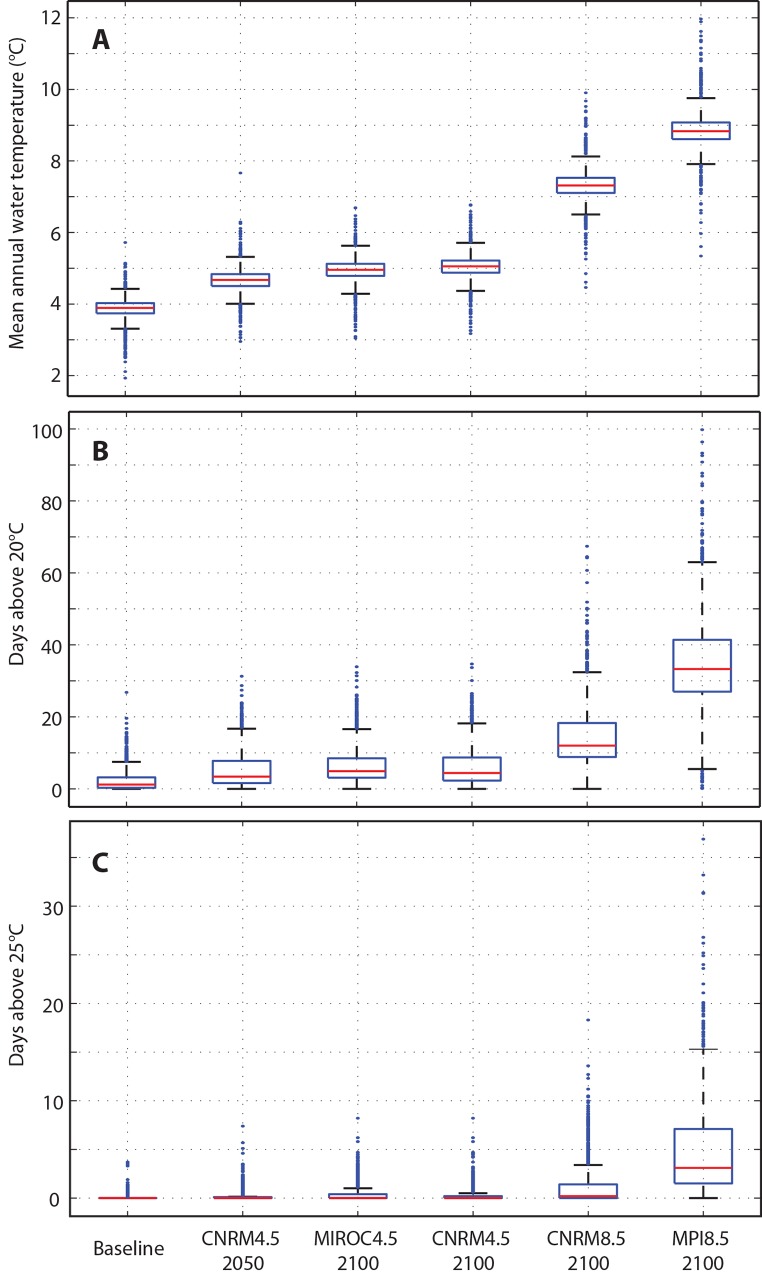
Summary of Temperature Data from All Model Results. A) Mean annual stream temperature, by model node, for each of the modeled scenarios. B) Average annual number of days above 20°C by model node, for each of the modeled scenarios. C) Average annual number of days above 25°C by model node, for each of the modeled scenarios. Box and whisker plots show distribution of average temperatures

Projected changes in mean annual stream temperatures generally mimic increases in air temperature, but increases in stream temperatures in all model runs are also modulated by groundwater inflows (Fig 8). For example, average annual air temperatures for the “cool” endmember model (MIROC5 RCP 4.5) increase by approximately 2.2°C by 2100 (Table 1), whereas average annual stream temperatures increase by only 1°C (Fig 8A; Fig 9A). In the “hot” endmember model (MPI-ESM-LR RCP 8.5) where we allowed groundwater temperatures to more closely track air temperatures, average air temperatures increase by nearly 8.5°C by 2100, but annual stream temperatures still increase by only 5°C (Fig 8B; Fig 9A). Thus, groundwater modulates stream temperatures to some degree regardless of our assumptions about how groundwater temperatures change in the future.

**Fig 10:**
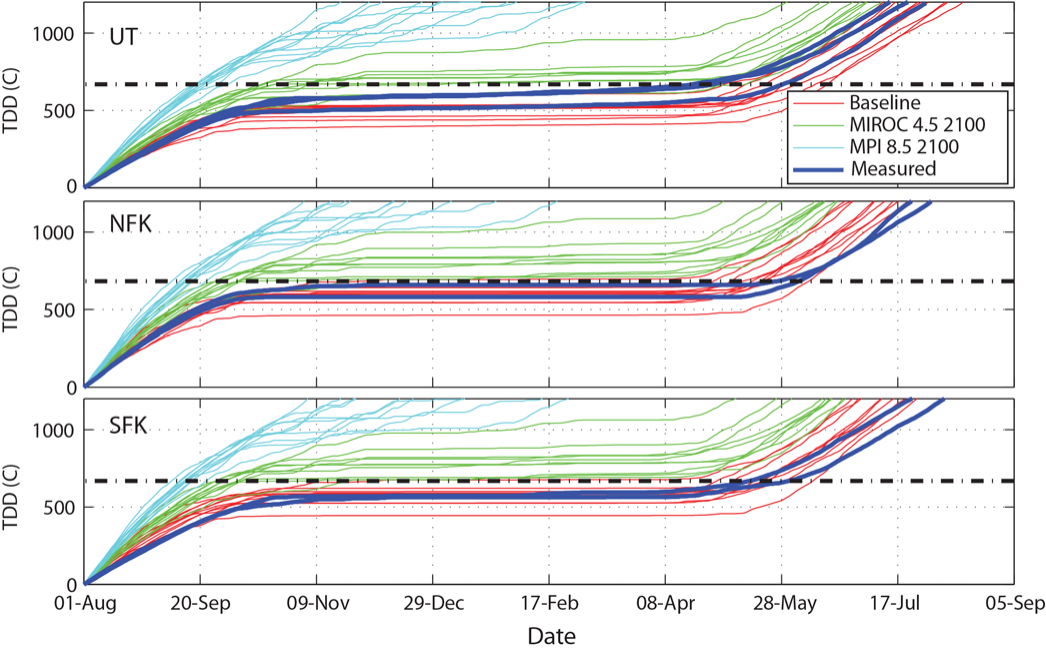
Comparison of Modeled TDD for Baseline vs. 2100 at the Three USGS Gages Assuming Lowest (MIROC, Green Line) and Highest (MPI, Cyan Lines) Future Temperature Scenarios. Thin colored lines show results from each of the 10 years in the simulations. Thick blue lines show cumulative TDD calculated from measured temperatures at the same sites. Black dash-dot lines represent estimated TDD required for sockeye to hatch [8].

Despite the modulating thermal effect of groundwater, simulated increases in average stream temperatures for both the “hot” and “cool” climate change scenarios indicate that salmon incubation time could be substantially altered in the future. Fig 10 shows the accumulation of TDD through the fall and winter months for baseline and future climate simulations, based on an assumed spawning date of August 1st. The simulated TDD trajectories under the baseline climate are very similar to observations at each of the three USGS gage sites, and predict a hatch date in early May. Under the “cool” future scenario, the 600 TDD threshold is exceeded within 2–3 months of spawning, whereas the “hot” model suggests that this threshold could be exceeded in less than 2 months. The exact duration of incubation may be difficult to estimate, since the 600 TDD threshold we have assumed here is based on controlled laboratory experiments, rather than field conditions [8]. Nonetheless, these simulation results indicate that salmon incubation is likely to be significantly altered under either of the endmember future climate scenarios.

**Fig 11:**
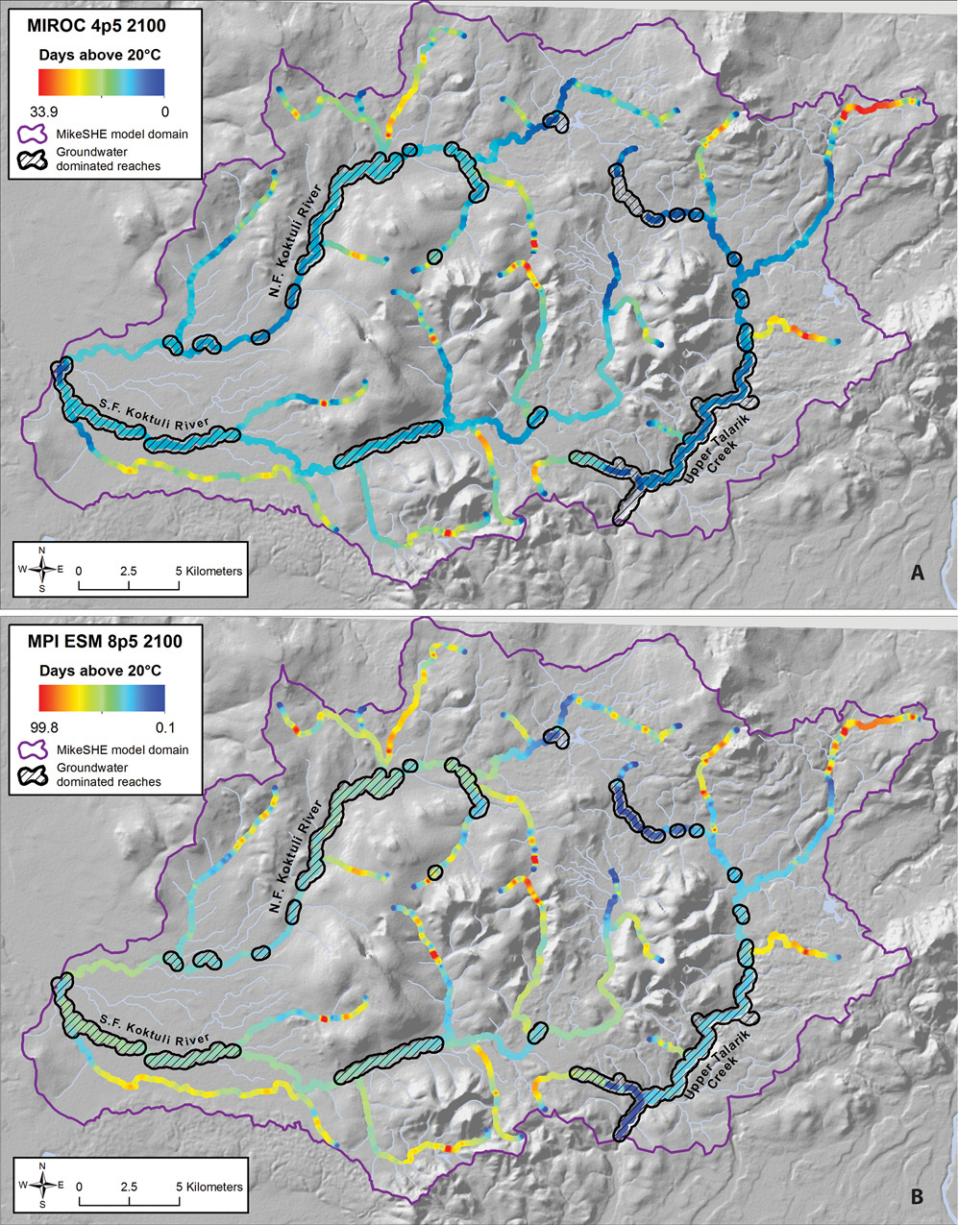
Modeled Number of Days Above 20°C in 2100 for (a) “Cool” Scenario (CNRM-CM5, RCP 4.5) and (b) “Hot” Scenario (MPI-ESM-8.5 RCP 8.5). Colored dots represent the average number of days above the threshold for each node in the model domain, averaged over the 10-year model run. Groundwater dominated reaches adapted from [15].

All of the RCP 4.5 scenarios result in an approximate doubling of the median number of days above 20°C, from approximately 2 days to approximately 5–6 days (Fig 9B). Under the higher RCP scenarios (CNRM 8.5 and MPI 8.5) the median number of days above 20°C increases to between ~15 and ~35. Even in some of the highest RCP scenarios, however, the moderating effects of higher flows, shading from vegetation, and groundwater inputs maintain suitable thermal habitat in many parts of the watershed for the majority of the year (Fig 11). All of the lower RCP scenarios indicate that the average number of days above the acute mortality threshold of 25°C remains at or near zero in the future climate runs; in the higher emissions scenarios this threshold is exceeded at least one day per year across much of the model domain (Fig 9C).

## Discussion

This study presents a spatially explicit and quantitative approach to estimating climate-mediated changes in ecological conditions in Bristol Bay. This is an essential tool in any ecosystem where climate and other proposed development activities could interact to compound ecological risk to sensitive receptors. While it may not be possible to perfectly characterize all of the details of this complex hydrologic system, this model closely matches the seasonal patterns of observed hydrographs, the timing of spring runoff, and seasonal variations in stream temperatures in the Nushagak and Kvichak headwaters. Climate changes projected from “hot” and “cold” endmember models each create gross changes in these baseline conditions that would significantly alter hydrologic regimes, and therefore habitat quality that supports this globally important salmon fishery [1]. The net response of salmonids to these projected changes is likely to be complex, and may be very difficult to predict. However, in the context of future mineral development in the region, it is clear that “baseline” conditions are not likely to be stationary.

Projected changes in flow are dominated by a change in the timing of peak annual runoff, which manifests itself in both increasing and more variable winter flows and the loss of the spring freshet. The magnitude of peak annual flows remains largely unchanged in our simulations, which suggests that the ability of the hydrologic system to flush out fine grained materials and replenish spawning and rearing habitat may be largely unchanged in the future. However, loss of the spring freshet could affect salmon in a variety of ways, including increasing the likelihood of predation for outmigrating salmon during lower flows, or causing smolts to arrive to nearshore environments before food sources are plentiful. Increasing and more variable winter flows could alter the balance between egg burial depths and scour depths [28], potentially resulting in more frequent scour of redds during incubation [26]. Depending on the magnitude of winter storm events, such changes have the potential to eliminate entire year classes of incubating eggs. Conversely, increases in average winter flows could increase available overwinter habitat, potentially benefiting salmon that do successfully emerge from fall and winter incubation.

The response of Bristol Bay salmon to increasing stream temperature is also likely to be complex. For example, a shorter incubation period due to warmer stream temperatures could result in fry emerging into a different hydrologic regime, where food sources or flow conditions may not be as suitable for rearing. Alternatively, emergence into warmer waters could speed juvenile growth rates, which could be a net benefit for survival for some species. An earlier emergence could also coincide with higher average flows and associated habitat, and potentially a period in which predation is lower. Scenarios such as these could potentially improve the chances of survival.

Increases in the occurrence of stream temperatures exceeding chronic and acute temperature stress levels are likely to be detrimental to salmonids in this system, but the degree of impact is also difficult to predict. For example, salmon might naturally migrate to refugia where stream temperatures are modulated by groundwater inputs and/or shading [33], as illustrated in Fig 11. In this case, climate change might simply shrink the availability of suitable habitat in this system. However, if juvenile salmon continue to occupy the upper reaches of tributaries where the highest temperatures are more likely to occur, warming stream temperatures could lead to increases in chronic and/or acute temperature stress.

Even if we could perfectly simulate future hydrologic regimes, the net response of the ecosystem to all of these interacting changes may be impossible to predict. However, it is clear from this study that the “baseline” hydrology of Bristol Bay is not static, a finding that must be incorporated into any decisions regarding proposed mineral development in the region. More than 600,000 acres of mining claims have been staked in these watersheds over the past two decades [39], and development of these resources could lead to additional hydrologic changes including habitat fragmentation, changes in the magnitude and timing of peak flows, and potentially the release of contaminants into downstream waters [3,4]. If the compounding effects of climate change and mineral development are not explicitly acknowledged, management strategies to mitigate potential mining effects may not be sufficiently protective of ecological resources. As an example, copper toxicity to salmon is modulated by other constituents in natural waters, including temperature [40,41]. Development of site-specific water quality criteria related to mining will need to explicitly acknowledge rising stream temperatures and other chemical changes in order to be protective. Including climate change into the mine permitting process could also fundamentally alter how other mitigation strategies are designed.

The expanse and diversity of lakes, streams and wetlands in the watersheds of Bristol Bay make this region one of the most productive and sustainable wild salmon fisheries in the world. Our study demonstrates that these hydrologic conditions are likely to change as temperatures rise over the next century, and will likely have significant influences on salmon in their freshwater environment. Throughout their evolutionary history salmon have been highly successful in adapting to changing conditions, and diversifying to take advantage of suitable habitat [42]. While the hydrologic and biological diversity of Bristol Bay is one of the key assets for adapting to changing climatic conditions, potential alteration and habitat loss associated with large-scale mining represents an additional risk factor for salmon that will interact with changes in temperature and stream flow associated with climate change [3]. Thus, assessment of ecological risk from potential large mines or other development scenarios will need to quantify and incorporate estimates of climate-induced risk as part of changing baseline conditions.

## Supporting Information

S1 File Simulated vs. Observed Hydrographs for Baseline Conditions at Each of the Stream Gaging Sites in the Model Domain (Figs A-N) and Simulated and Measured Stream Temperatures at USGS Gaging Sites (Figs O-P). USGS North Fork Koktuli Gage in Lower North Fork Koktuli (**Fig A**). NK100C in Upper North Fork Koktuli (**Fig B**). NK119A in Upper North Fork Koktuli **(Fig C**). USGS South Fork Koktuli Gage in Middle South Fork Koktuli **(Fig D**). SK100A Gage in Lower South Fork Koktuli, Near Junction with North Fork Koktuli (**Fig E**). SK100B1 in Middle South Fork Koktuli (**Fig F**). SK100C in Middle South Fork Koktuli (**Fig G**). SK100F in Upper South Fork Koktuli (**Fig H**). SK100G in Upper South Fork Koktuli (**Fig I**). SK119A, a Tributary to the Middle South Fork Koktuli (**Fig J**). SK124A, a Tributary to the Middle South Fork Koktuli (**Fig K**). UT100D in the Upper Upper Talarik Drainage (**Fig L**). UT100E in Uppermost Upper Talarik Creek (**Fig M**). UT119A, a Tributary to the Lower Upper Talarik (**Fig N**). Temperatures at USGS North Fork Koktuli Gage Site (**Fig O**). Temperatures at USGS South Fork Koktuli Gage Site (**Fig P**).
(DOCX)
