# Correction: Hydrologic Alterations from Climate Change Inform Assessment of Ecological Risk to Pacific Salmon in Bristol Bay, Alaska

**DOI:** 10.1371/journal.pone.0147242

**Published:** 2016-01-13

**Authors:** Cameron Wobus, Robert Prucha, David Albert, Christine Woll, Maria Loinaz, Russell Jones, Constance Travers

Dr. Constance Travers should be included in the author byline instead of the Acknowledgments. He should be listed as the seventh author, and his affiliation is #1: Abt Associates, Boulder, Colorado, United States of America. The contributions of this author are as follows: Conceived and designed the experiments, analyzed the data, and wrote the manuscript.
