# Supplementary material for: Hydrologic Alterations from Climate Change Inform Assessment of Ecological Risk to Pacific Salmon in Bristol Bay, Alaska
Source: PLoS One. 2015 Dec 8;10(12):e0143905. doi: 10.1371/journal.pone.0143905 (PMC4672932; doi:10.1371/journal.pone.0143905)
Supplement: S1 File — USGS North Fork Koktuli Gage in Lower North Fork Koktuli (Fig A). NK100C in Upper North Fork Koktuli (Fig B). NK119A in Upper North Fork Koktuli (Fig C). USGS South Fork Koktuli Gage in Middle South Fork Koktuli (Fig D). SK100A Gage in Lower South Fork Koktuli, Near Junction with North Fork Koktuli (Fig E). SK100B1 in Middle South Fork Koktuli (Fig F). SK100C in Middle South Fork Koktuli (Fig G). SK100F in Upper South Fork Koktuli (Fig H). SK100G in Upper South Fork Koktuli (Fig I). SK119A, a Tributary to the Middle South Fork Koktuli (Fig J). SK124A, a Tributary to the Middle South Fork Koktuli (Fig K). UT100D in the Upper Upper Talarik Drainage (Fig L). UT100E in Uppermost Upper Talarik Creek (Fig M). UT119A, a Tributary to the Lower Upper Talarik (Fig N). Temperatures at USGS North Fork Koktuli Gage Site (Fig O). Temperatures at USGS South Fork Koktuli Gage Site (Fig P). (DOCX) [file pone.0143905.s001.docx]

**Hydrologic alterations from climate change inform assessment of ecological risk to Pacific Salmon in Bristol Bay, Alaska**

Cameron Wobus^1^*, Robert Prucha^2^, David Albert^3^, Christine Woll^3^, Maria Loinaz^4^, Russell Jones^1^

**Supplemental Information File**

Figures A through N: Simulated vs observed hydrographs for baseline conditions at each of the stream gaging sites in the model domain


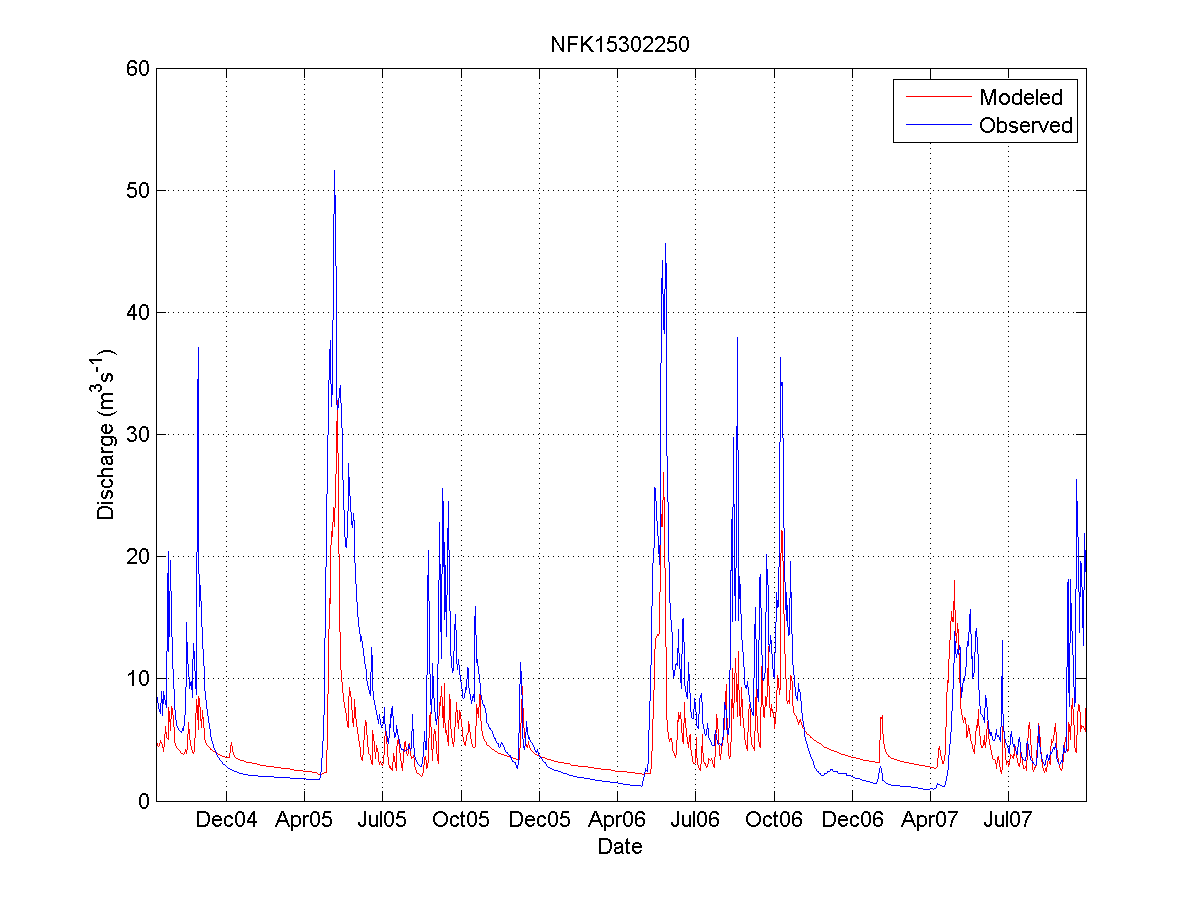


Figure A. USGS North Fork Koktuli Gage in lower North Fork Koktuli


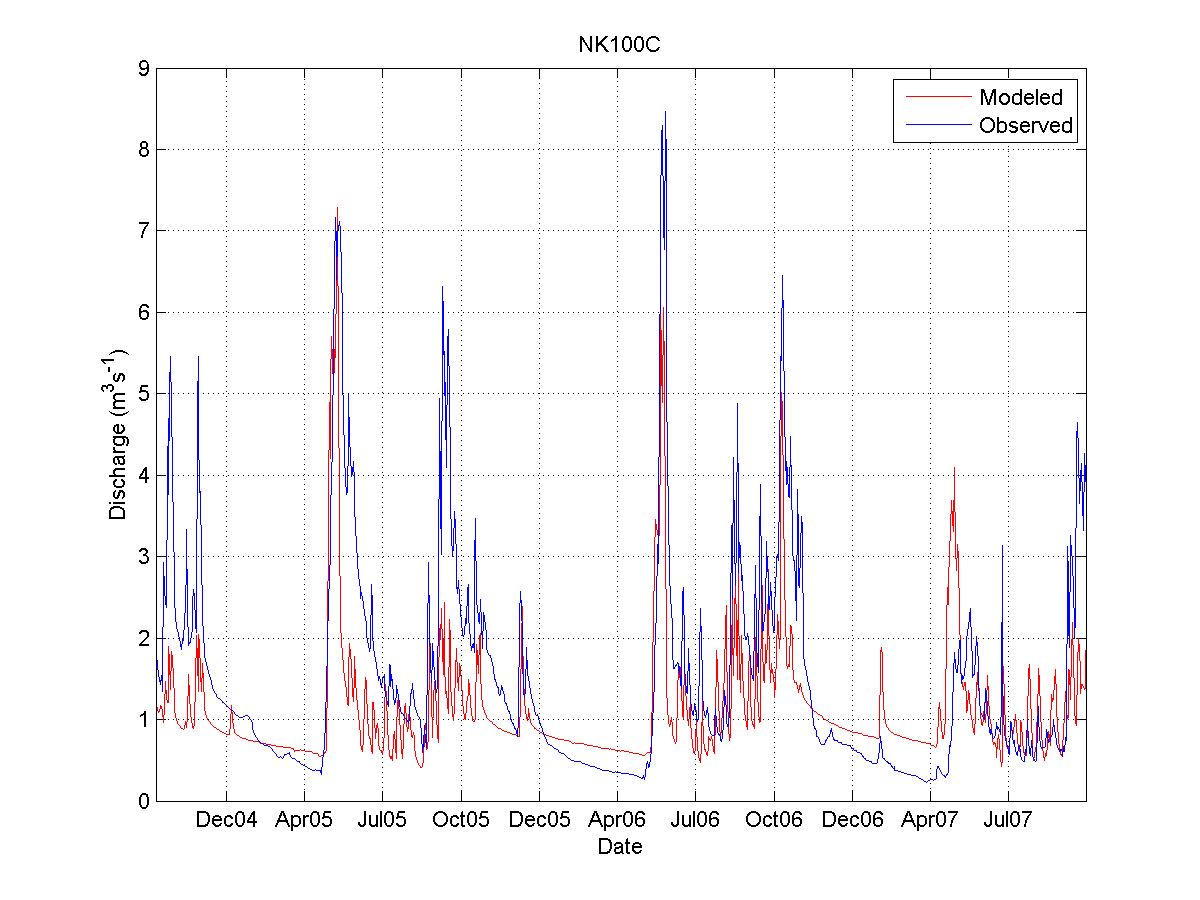
Figure B. NK100C in upper North Fork Koktuli
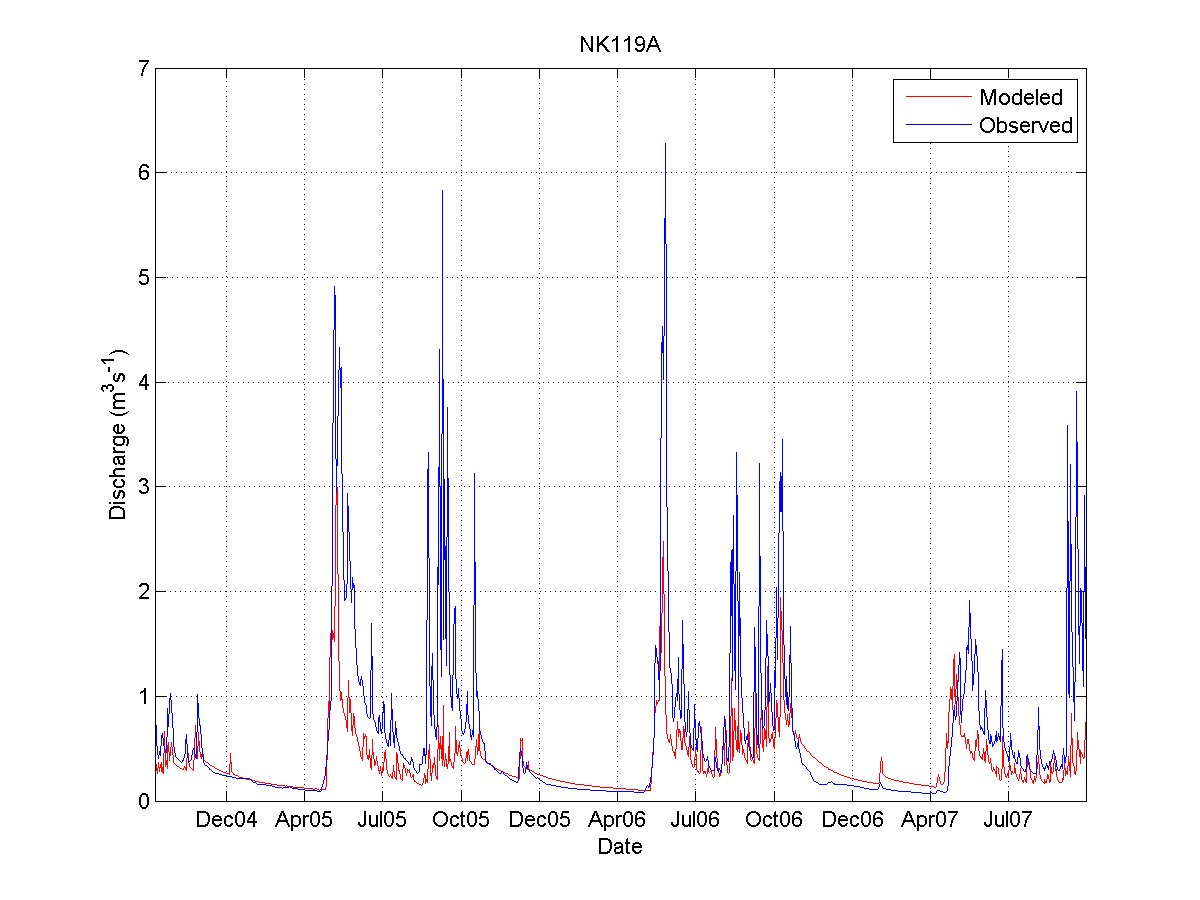
 Figure C. NK119A in upper North Fork Koktuli
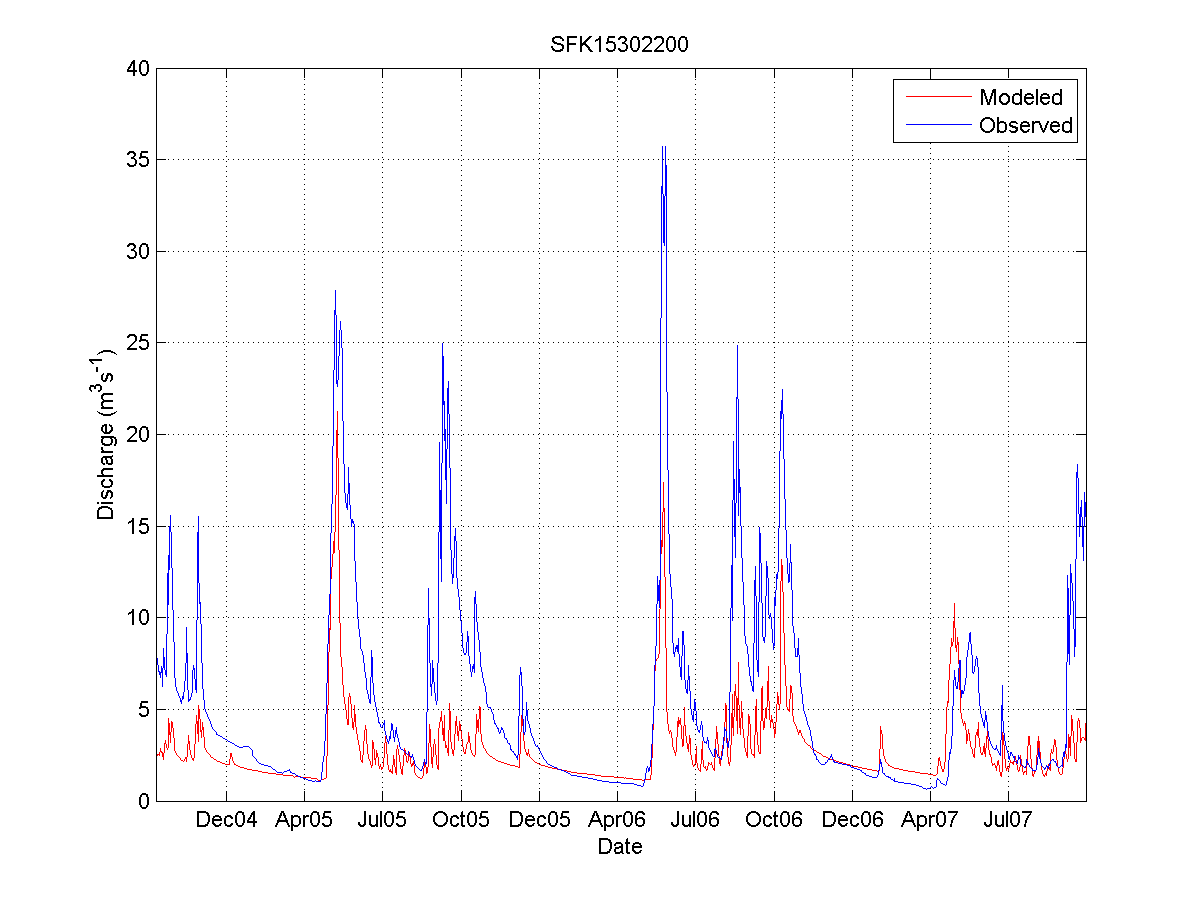
 Figure D. USGS South Fork Koktuli gage in middle South Fork Koktuli
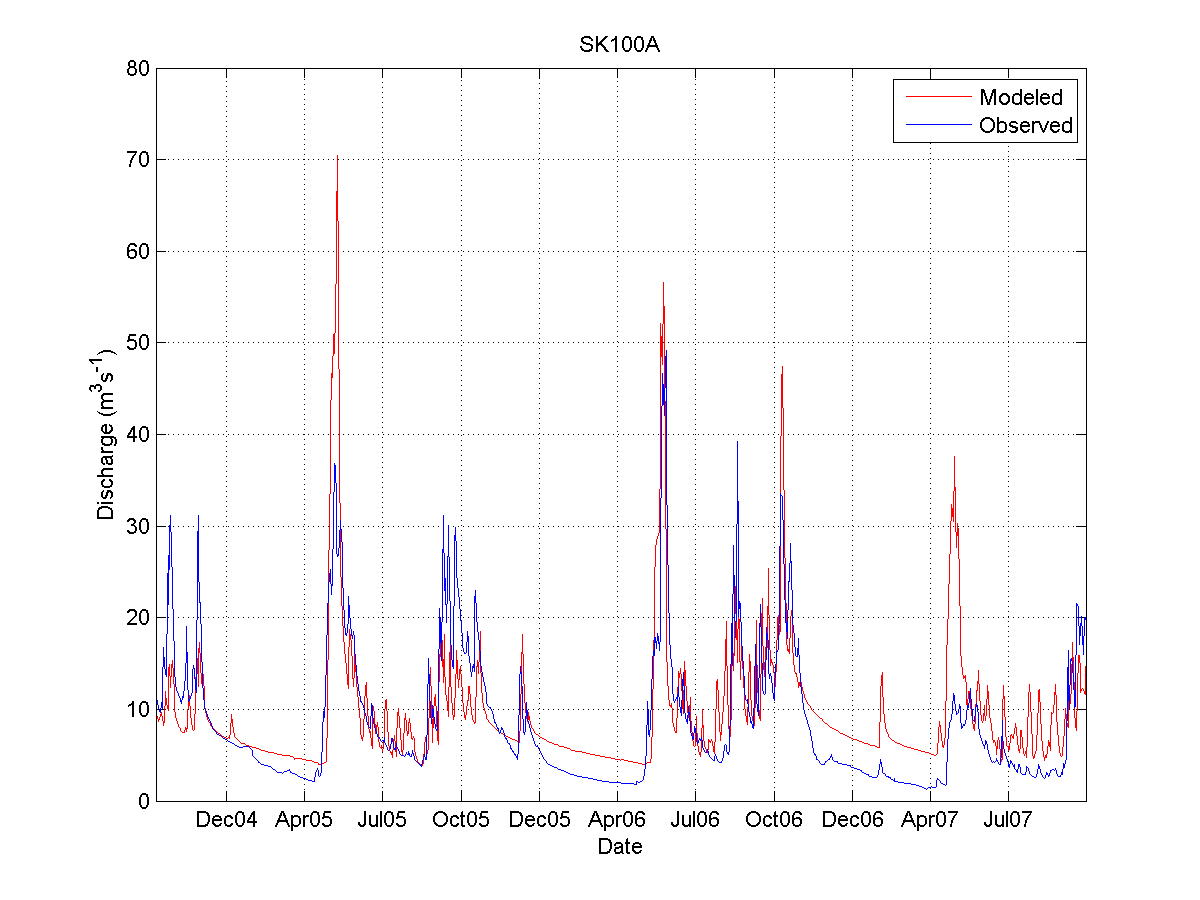
 Figure E. SK100A gage in lower South Fork Koktuli, near junction with North Fork Koktuli
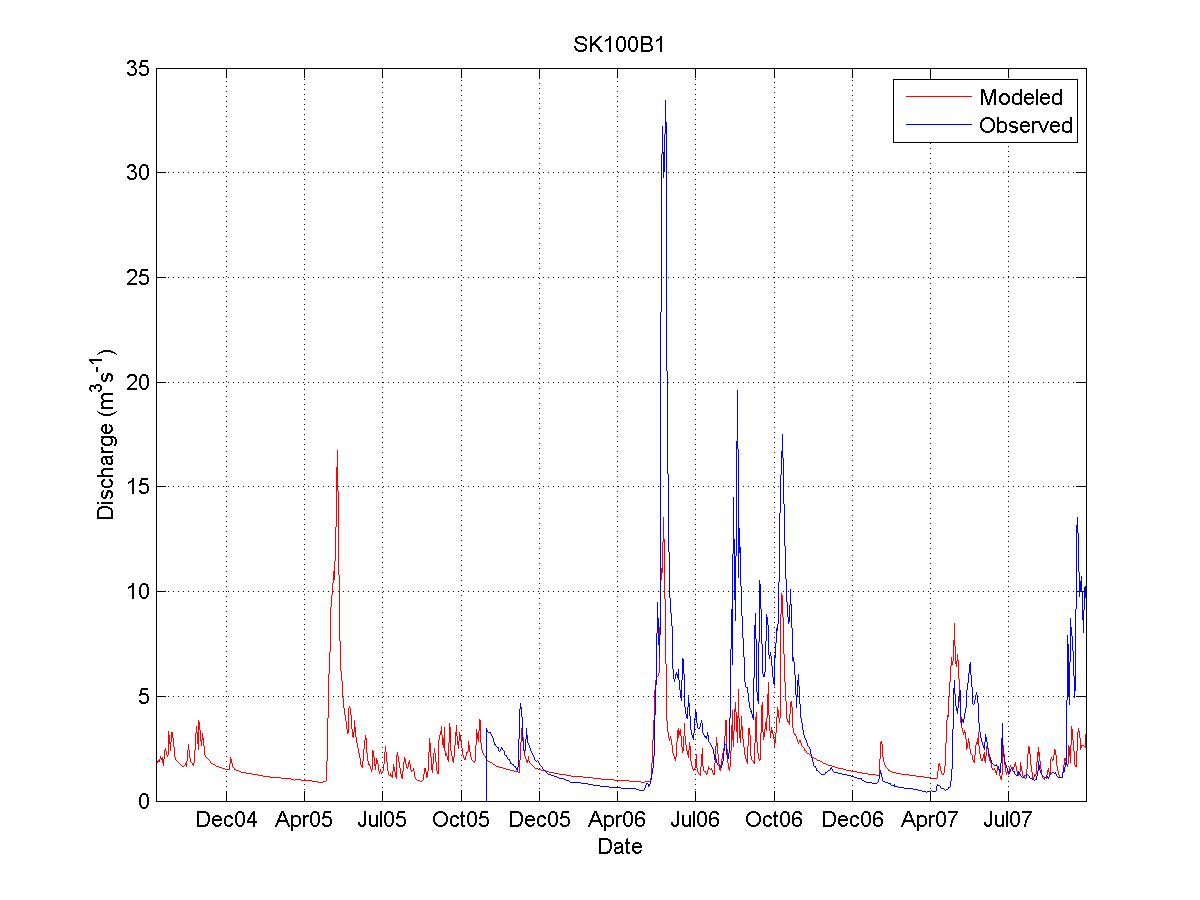
 Figure F. SK100B1 in middle South Fork Koktuli
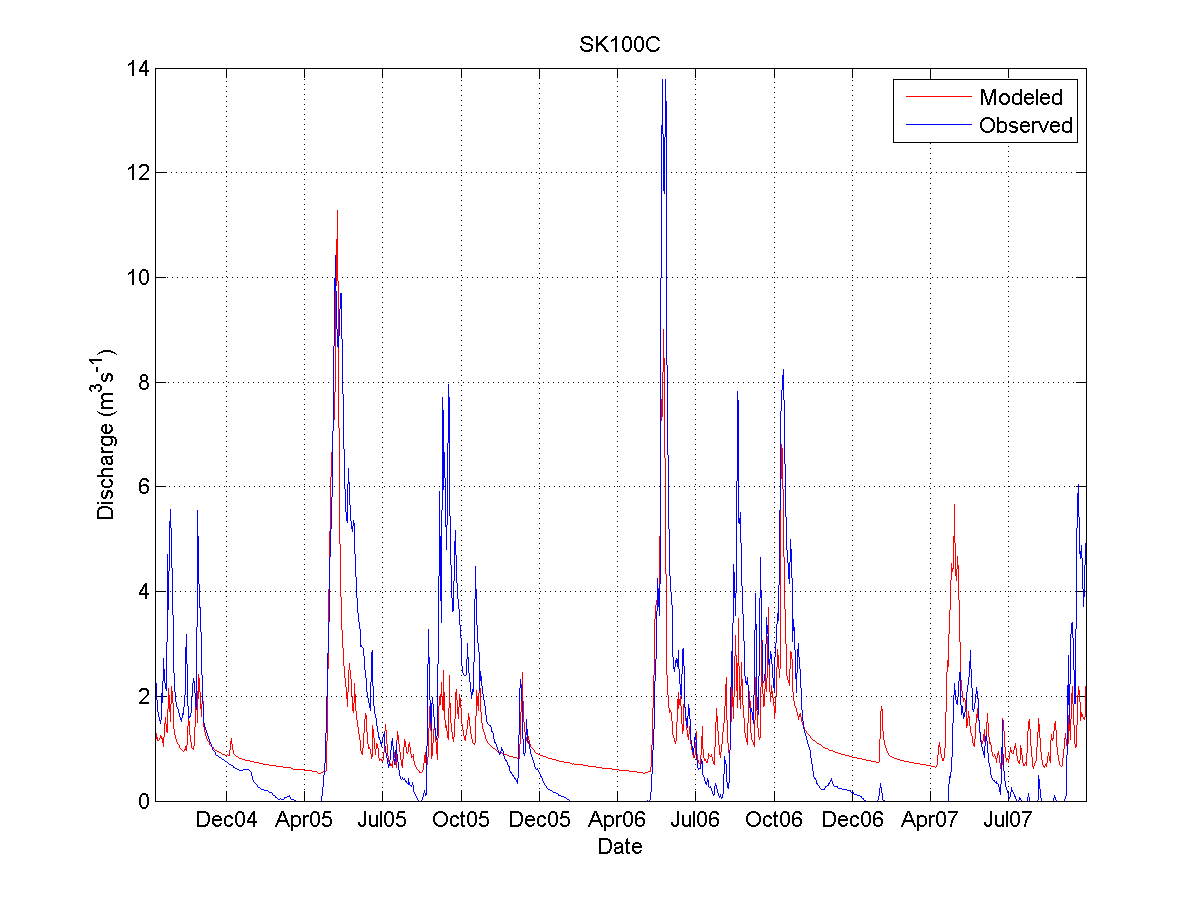
 Figure G. SK100C in middle South Fork Koktuli
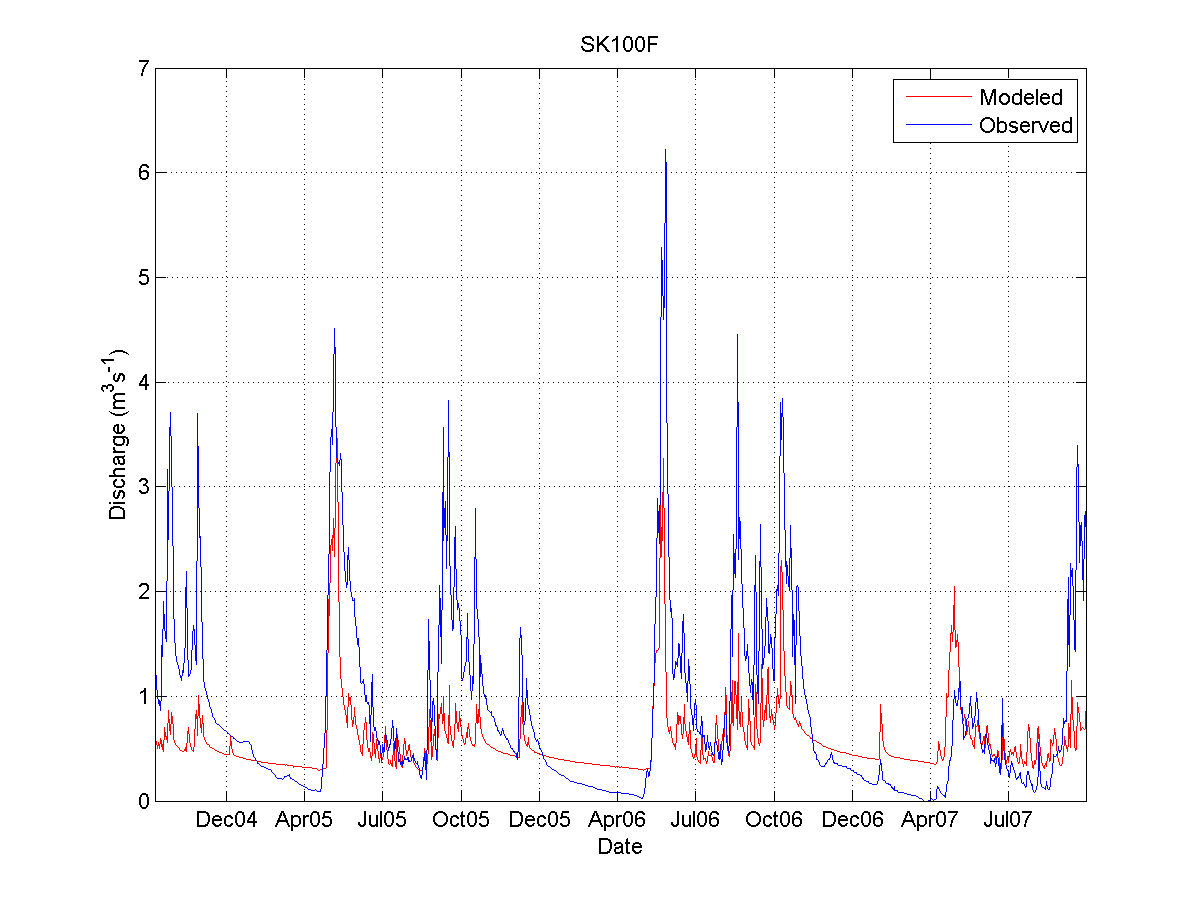
 Figure H. SK100F in upper South Fork Koktuli
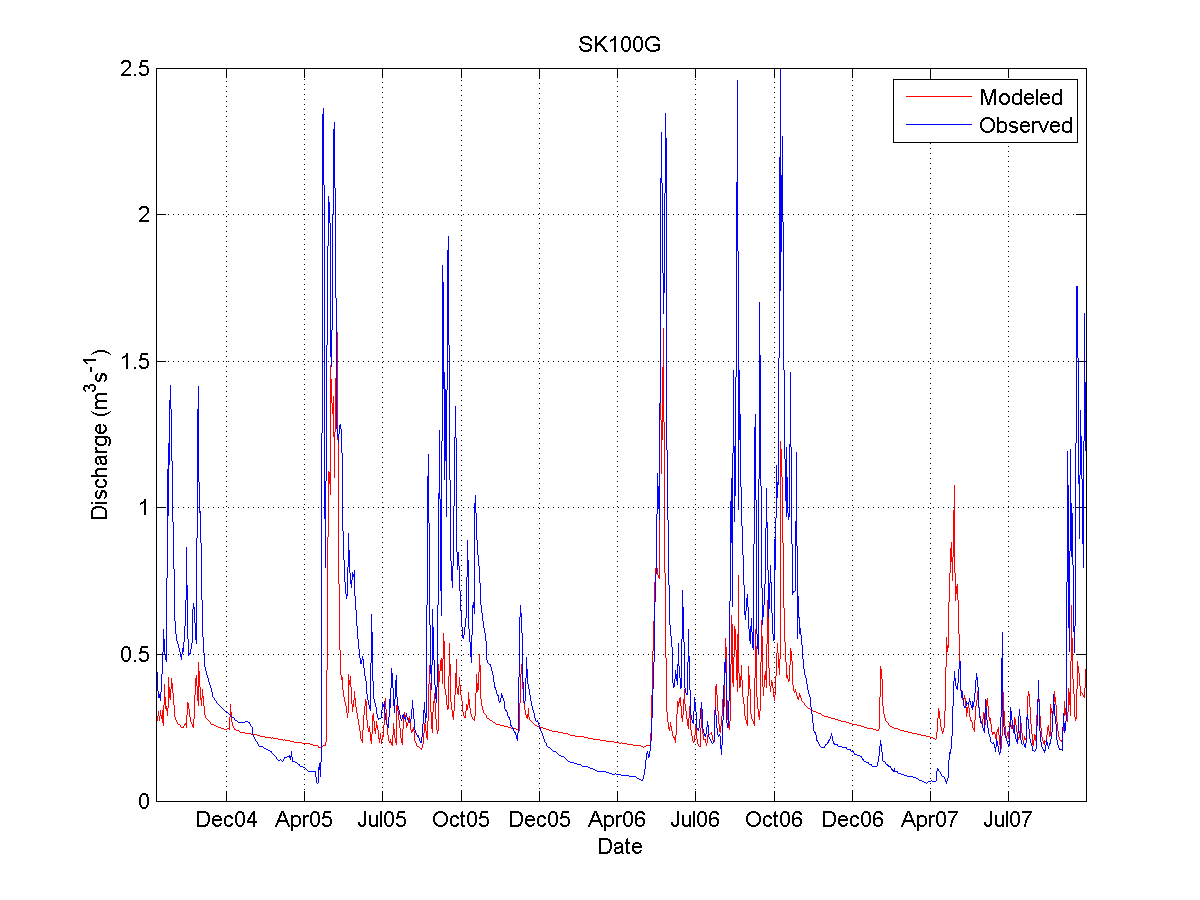
 Figure I. SK100G in upper South Fork Koktuli
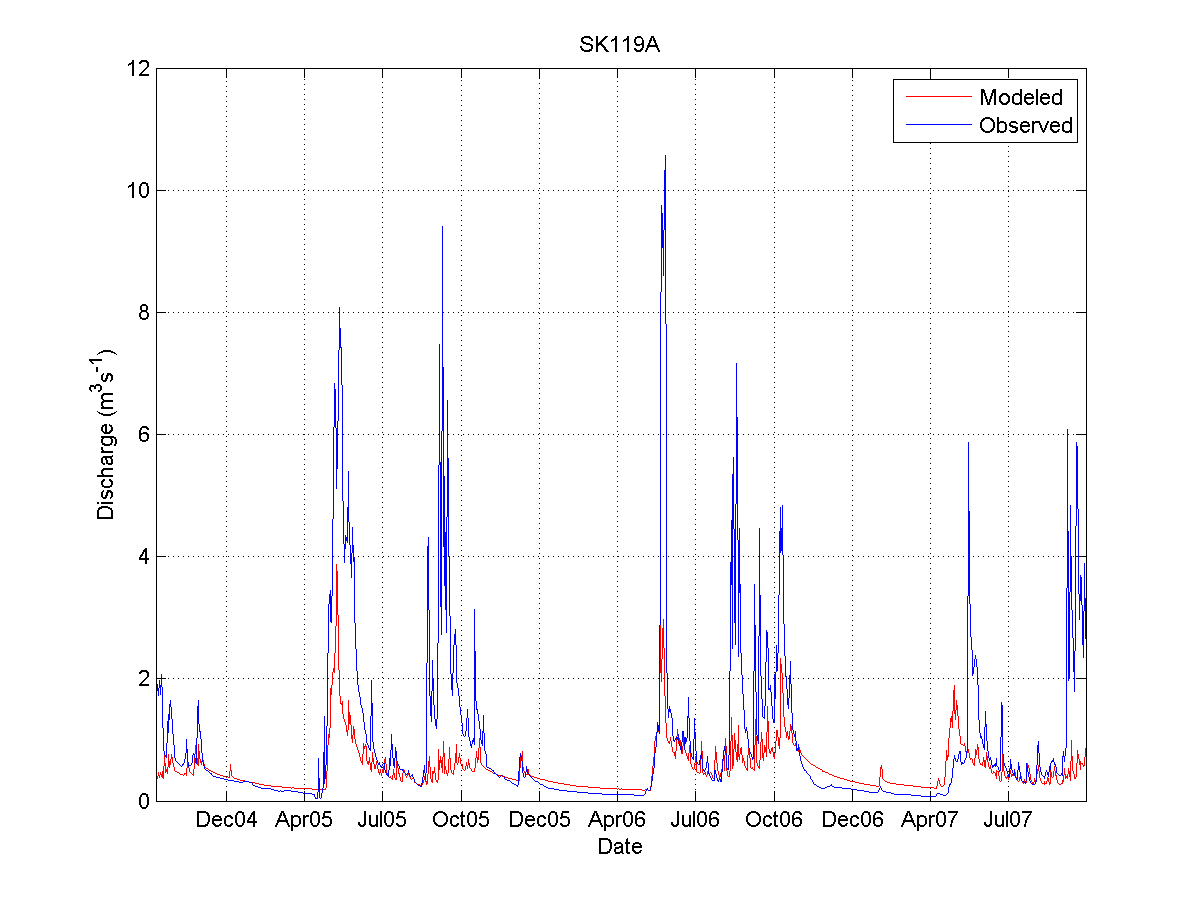
 Figure J. SK119A, a tributary to the middle South Fork Koktuli
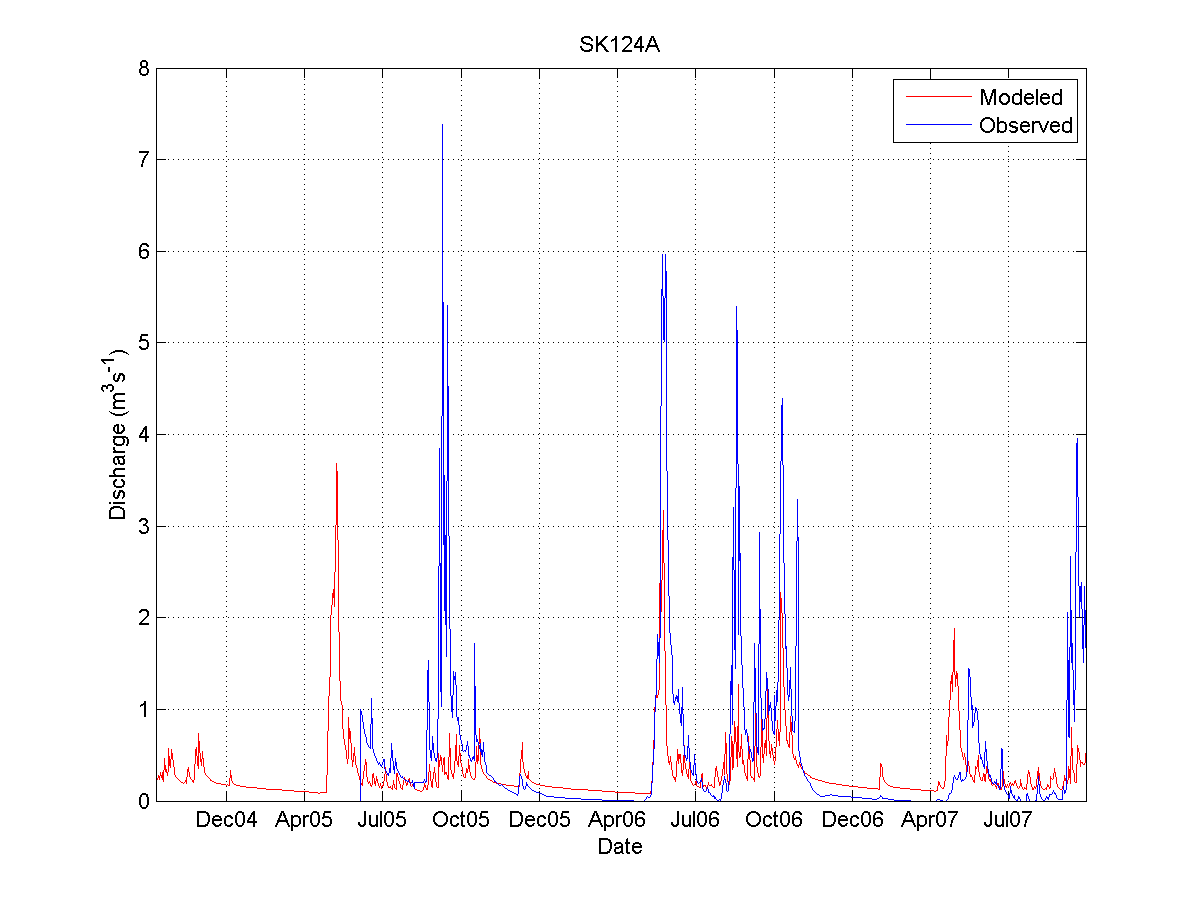
 Figure K. SK124A, a tributary to the middle South Fork Koktuli
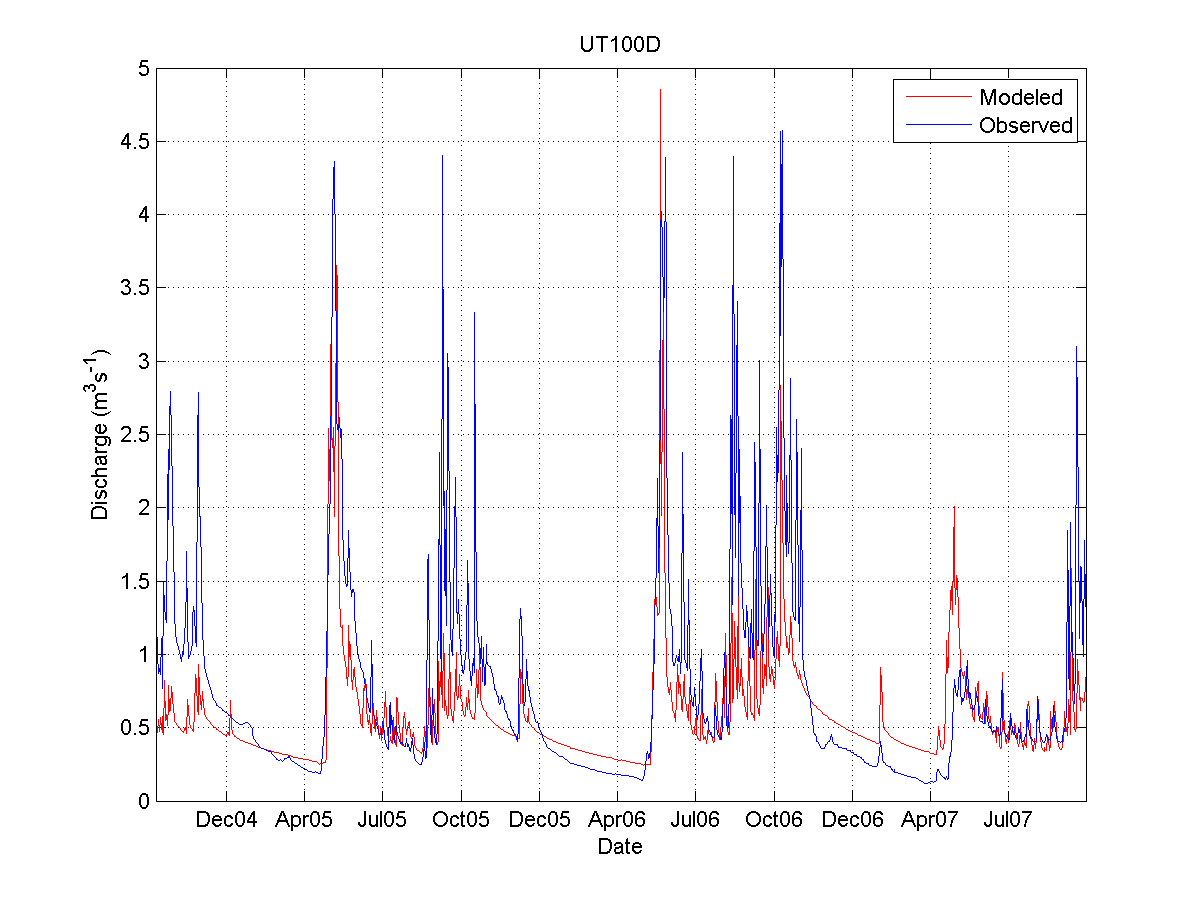
 Figure L. UT100D in the upper Upper Talarik drainage
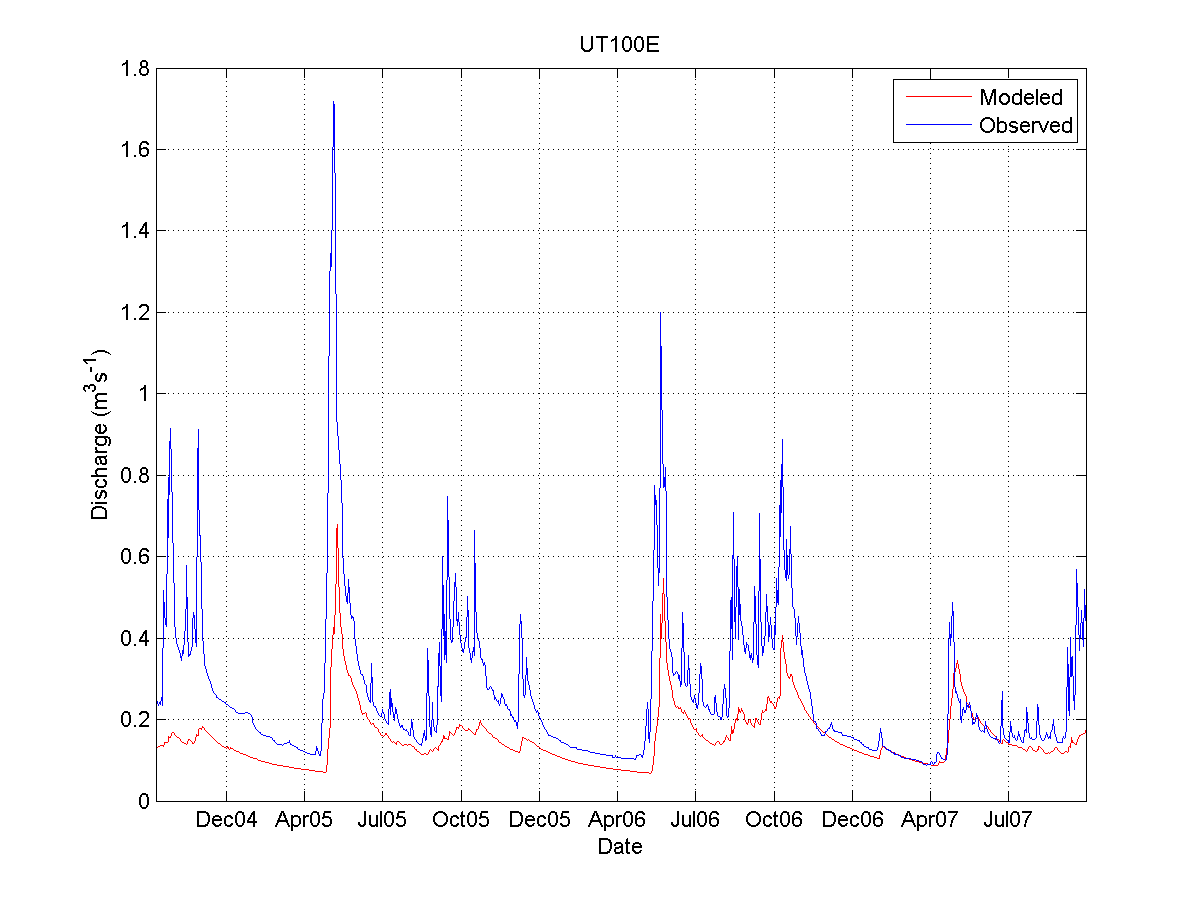
 Figure M. UT100E in uppermost Upper Talarik Creek.
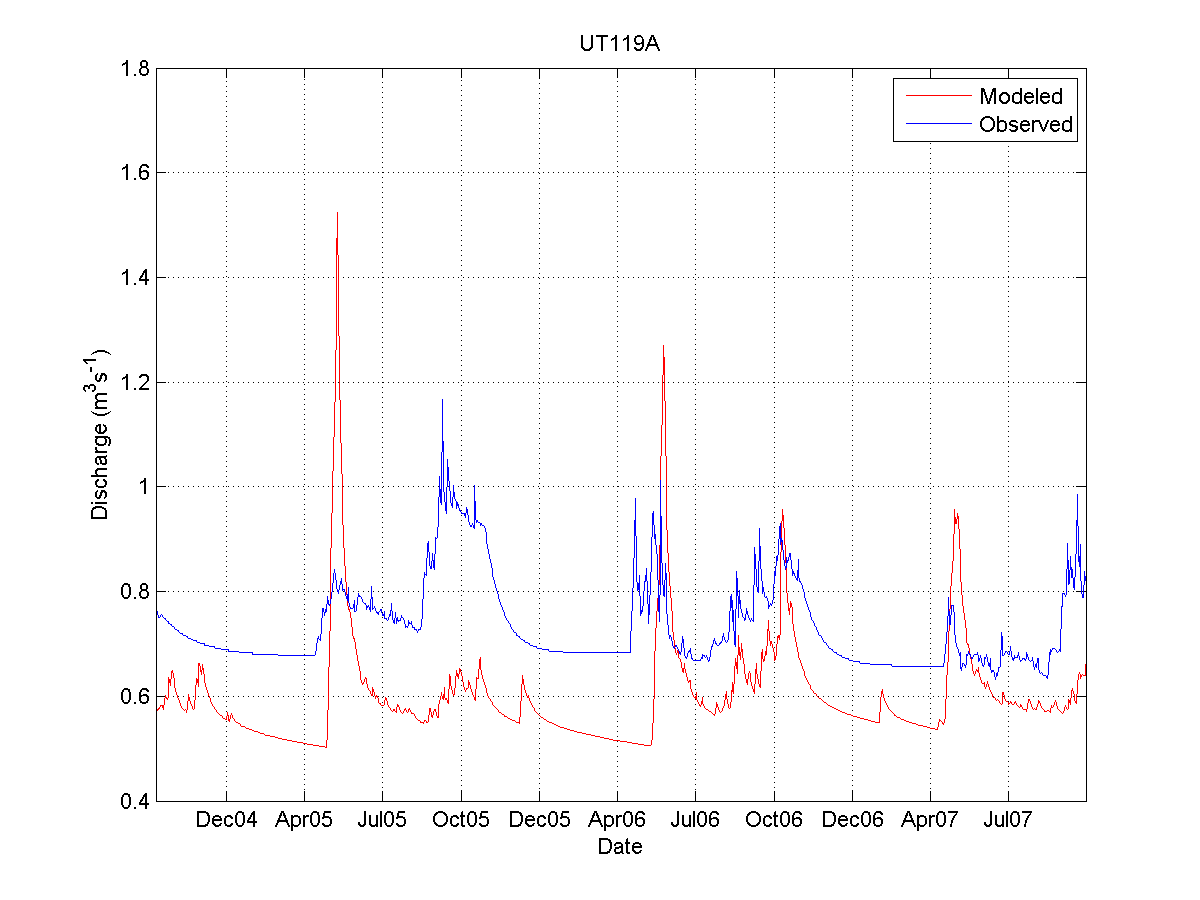
 Figure N. UT119A, a tributary to the lower Upper Talarik

Figures O and P. Simulated and measured stream temperatures at USGS gaging sites


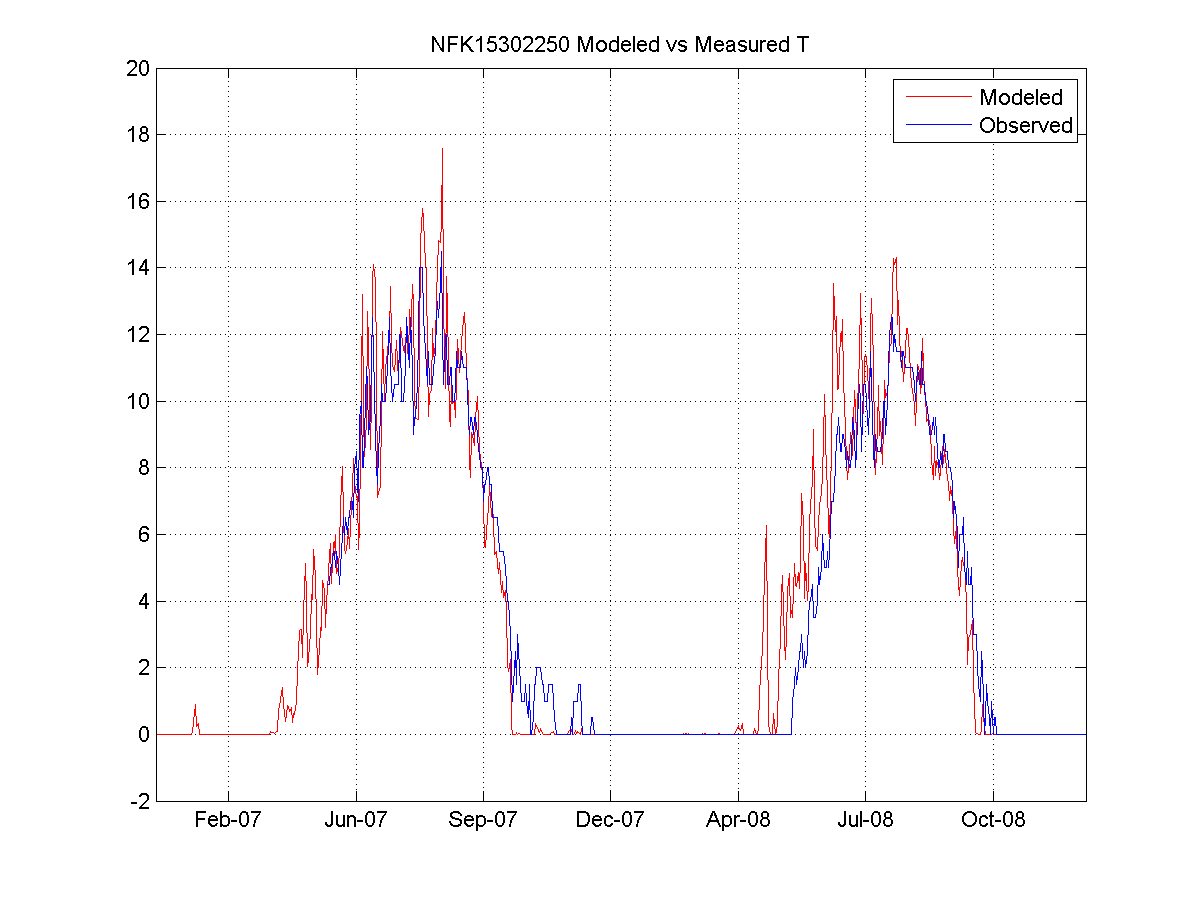
Figure O. Temperatures at USGS North Fork Koktuli gage site


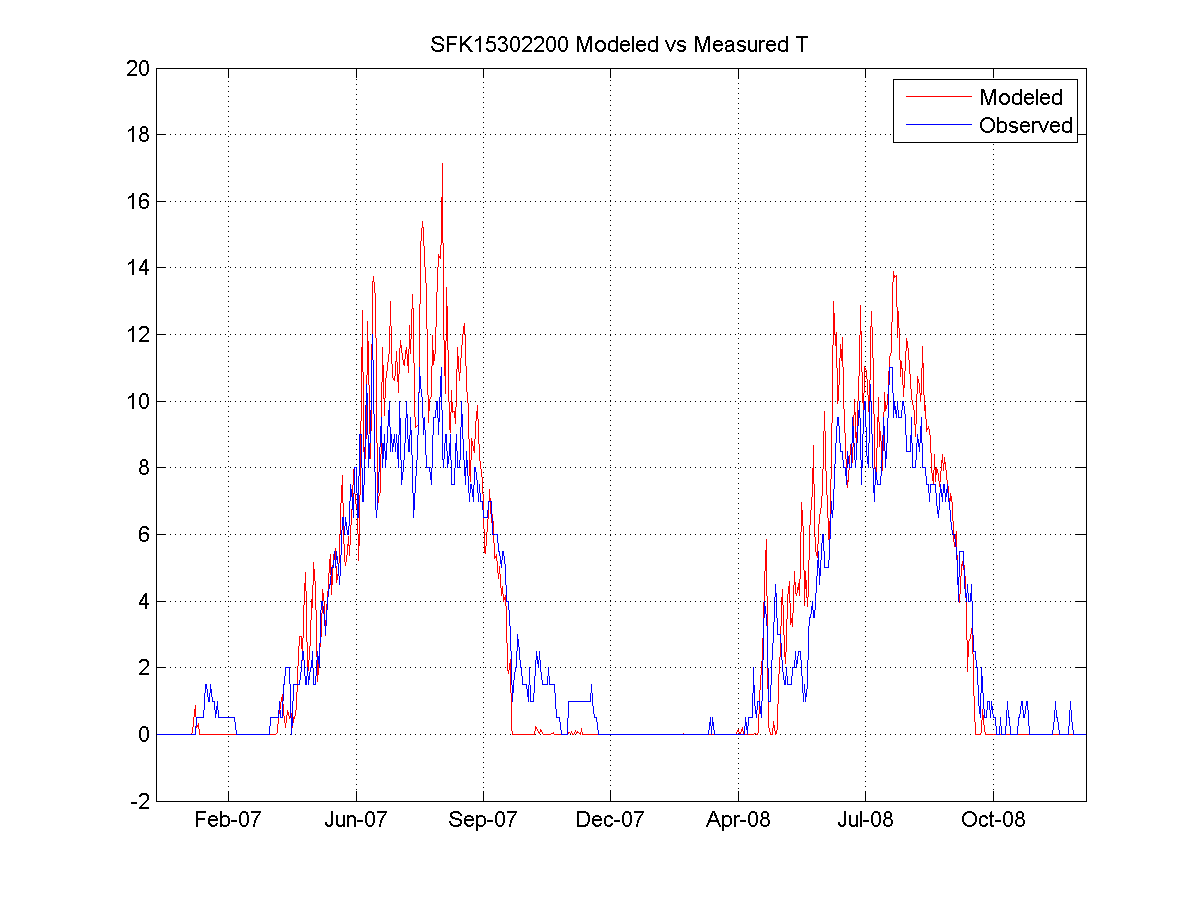
 Figure P. Temperatures at USGS South Fork Koktuli gage site
